# A mechanism-based model of immune status effects on antibiotic PK/PD targets in bacteremia

**DOI:** 10.1007/s10928-026-10063-6

**Published:** 2026-09-22

**Authors:** Marinda van de Kreeke, Anh Duc Pham, Michelle Mehciz, Tamara Jordens, Marnix G. Uyterlinde, Rob C. van Wijk, Linda B. S. Aulin, Catherijne A. J. Knibbe, Elke H. J. Krekels, Laura B. Zwep, J. G. Coen van Hasselt

**Affiliations:** 1 Division of Systems Pharmacology and Pharmacy, Leiden Academic Centre for Drug Research, Leiden, The Netherlands; 2 Department of Clinical Pharmacy, St. Antonius Hospital, Nieuwegein, The Netherlands; 3 Certara Inc, Radnor, PA USA

**Keywords:** Mathematical modeling, Neutrophils, Monocytes, Antibiotic dosing

## Abstract

A key determinant of antibiotic dose selection is the pharmacokinetic/pharmacodynamic (PK/PD) target, typically determined using dose fractionation studies in preclinical infection models. However, such studies often do not consider the host immune response, despite its important role in bacterial infection. We therefore aimed to systematically characterize the potential contributions of the innate immune response on antibiotic PK/PD targets using a novel mathematical mechanism-based model, incorporating interactions between neutrophils, monocytes and bacterial pathogens. We parametrized the model using data from multiple *in vitro* host-pathogen interaction studies and calibrated using data from previous *in vivo* infection models. Key parameters included phagocytosis and digestion rates of neutrophils and monocytes, driven by immune cell concentrations and finite ingestion capacities. To describe data from immune competent rodent infection models, maximum phagocytosis rates required a 77% reduction relative to *in vitro* estimates for both cell types. The calibrated host-pathogen interaction model was then integrated with a pharmacodynamic model accounting for pathogen-drug interactions for four antibiotic modalities. We performed *in silico* dose fractionation studies for bacteremia thereby evaluating the impact of different types and magnitudes of immune deficiencies, e.g., neutropenia, monocytopenia, on PK/PD targets in humans. The combination of severe neutropenia and monocytopenia required up to 2.2-fold increase in target AUC/MIC for concentration-dependent antibiotics, and up to a 58% increase in T>MIC for time-dependent antibiotics, compared with immune competent individuals. In conclusion, this study provides general insights into the impact of neutrophil and monocyte suppression on PK/PD targets and associated dosage adjustments in case of immune suppression.

**Supplementary Information:**

The online version contains supplementary material available at https://doi.org/10.1007/s10928-026-10063-6.

## Introduction

Antibiotic dosing guidelines are primarily based on the relationship between antibiotic exposure and the *in vitro* minimum inhibitory concentration (MIC) of specific bacterial strains, which is described by the pharmacokinetic/pharmacodynamic (PK/PD) indices [1, 2]. PK/PD indices are typically derived using *in vivo* dose fractionation studies in immune suppressed animal models, and as such, do not explicitly consider the impact of immune status [3]. Even though dosing guidelines do provide some guidance on antibiotic choice and dosing in patients with altered immune status, these recommendations are typically based on limited evidence. Therefore, there exists uncertainty about how therapeutic targets should be adjusted for patients with varying degrees of immune suppression, such as in individuals with neutropenia or monocytopenia, and for which antibiotic treatments are more often associated with treatment failure [4–7]. Further understanding in the contribution of immune status on antibiotic PK/PD indices is therefore of relevance to guide how antibiotic dosing may need to be adjusted in patients with altered immune status.

In immune competent hosts, circulating innate immune cells often clear pathogenic bacteria rapidly, limiting progression to more severe conditions such as bacteremia [8]. However, in case of an immune suppressed status, for example in patients undergoing myelosuppressive chemotherapy, the risk of persistent bacteremia and sepsis is significantly increased [9]. The innate immune response plays a critical role in clearing bacteria, especially during the initial hours before the adaptive immune system becomes active [10, 11]. Neutrophils play a central role in the innate immune response as primary effector cells in early host defense. Monocytes are a second key type of immune cells involved in early pathogen defense, and together with neutrophils, they eliminate pathogens through phagocytosis and digestion [12, 13]. In turn, bacteria influence the lifespan and turnover of these immune cells [14]. Overall, this highlights the complex interplay between bacteria and the host immune system, raising the question how such host-pathogen interactions during immune suppression may impact antibacterial PK/PD indices and ultimately dosing strategies.

Mathematical modeling represents a relevant strategy to characterize the interactions between host, pathogen and antibiotics in the context of antibiotic therapy. Mathematical models for antibiotic PKPD are routinely used to guide antibiotic dose regimen optimization. In addition, various mathematical models have been reported which demonstrate their utility in understanding interactions between host immunity and bacterial infection [15–24]. These models typically centered on localized infections and not bacteremia. Moreover these models primarily focused on the role of immune cell-mediated bacterial interactions, excluded monocytes, and, typically did not address the effects of host-pathogen interactions on antibiotic PKPD.

In the current study we aimed to assess how changes in immune status may affect antibiotic PK/PD targets, for different types of antibiotic agents, with a focus on early treatment of bacteremia. To this end, we developed a novel mathematical modeling framework capturing bacteria-host-drug interactions, accounting for early-stage interactions between bacterial pathogens, neutrophils and monocytes, as well as antibiotic PKPD relationships. Using *in silico* dose-fractionation studies we then evaluated how PK/PD targets may be affected when changes in innate immune cell concentrations occur.

## Methods

A mechanism-based model was designed to incorporate key early host immunity-pathogen interactions of importance for pathogen clearance, including phagocytosis and subsequent digestion by neutrophils and monocytes, parameterizing the model based on data primarily derived from various *in vitro* studies. Key model parameters to which bacterial clearance was found sensitive, were identified in a global sensitivity analysis. Subsequently, the model was calibrated to *in vivo* data from immune competent mice by adapting the key model parameters. The impact of different types of hypothetical antibiotic agents was then evaluated by integrating the mechanism-based model with a PKPD model component. Finally, the model was applied for *in silico* dose fractionation simulation studies to derive PK/PD targets under varying degrees of immune deficiency, assessing the relationship between immune function, antibiotic exposure, and bacterial clearance.

### Mechanism-based model development

#### Model description

The structure of the mechanism-based model focused on key host-pathogen interactions related the early response to bacterial infections relevant for bacterial clearance, and included phagocytosis and digestion of bacteria by neutrophils and monocytes, and the influence of bacteria on the death rate of neutrophils, and is described below.

Bacterial concentrations (B) over time were described with a net growth rate (*kg*), which was capacity limited (*Bmax*), and by the phagocytic effects of neutrophils ($k_{np}$) and monocytes ($k_{mp}$) (Eq. 1).

1 $$\begin{aligned} \frac{d}{dt}(B) = kg \cdot B \cdot (1 - \frac{B}{Bmax}) - k_{np} \cdot B - k_{mp} \cdot B \end{aligned}$$

Phagocytosis and digestion rates ($k_{ix}$) of immune cells were described with a dependency on immune cell concentrations. This dependency was described by an Emax function where hill was set to 1 (Eq. 2). Secondly, an additional effect of phagocytosis and digestion capacity of the immune cells was estimated. Relationships that were evaluated to describe this influence included functions with an estimated *slope* and a power (*p*) that was either 1 or estimated (Eq. 3) or sigmoidal functions in which the ratio leading to half the maximum process rate ($(B_I/I)_{x50}$), and the hill parameter (*γ *) (Eq. 4) were estimated. When insufficient data were available to estimate separate parameter values, parameters were assumed to be equal between neutrophils and monocytes.

2 $$\begin{aligned} k_{ix} = k_{ix,max} \cdot \left( \frac{I}{I_{x50} + I} \right) \end{aligned}$$

3 $$\begin{aligned} k_{ix} = k_{ix,max} \cdot \left( \frac{I}{I_{x50} + I} \right) - \left( slope \cdot (B_I/I)^{p} \right) \end{aligned}$$

4 $$\begin{aligned} k_{ix} = k_{ix,max} \cdot \left( \frac{I}{I_{x50} + I} \right) \cdot \left( 1 - \frac{(B_I/I)^\gamma }{(B_I/I)_{x50}^\gamma + (B_I/I)^\gamma } \right) \end{aligned}$$

Here, $k_{ix}$ is the rate *k* of a process *x* (i.e., phagocytosis or digestion) for a specific immune cell *i* (i.e., neutrophils or monocytes). $k_{ix,max}$ is the maximum process rate, *I* is immune cell concentration, $I_{50}$ is the immune cell concentration leading to half the maximum process rate, $B_I$ is the intracellular bacterial concentration, defined as the concentration of bacteria within immune cells.

The decay of neutrophil and monocyte concentrations over time was first described mechanistically, if data were available, with a combination of natural apoptosis ($k_{i, apop}$) and phagocytosis-induced cell death ($k_{i,picd}$) (Eq. 5), based on *in vitro* experiments where immune cells slowly disappear over time but do not grow.

5 $$\begin{aligned} \frac{d}{dt}(I) = - k_{i,apop} \cdot I - k_{i,picd} \cdot I \end{aligned}$$

However, when no data were available to estimate these parameters, an empiric decay function was applied to account for degradation of phagocytic and/or digestion activity over time (Eq. 6).

6 $$\begin{aligned} k_{ix,decay} = k_{ix} \cdot e^{-k_{decay,ix} \cdot t} \end{aligned}$$

Here, $k_{ix,decay}$ is the rate *k* of a process *x* (i.e., phagocytosis or digestion) for a specific immune cell *i* (i.e., neutrophils or monocytes) and scaled by $k_{decay,ix}$, the decay rate constant for a specific immune cell and process, and *t* is time.

The host-pathogen interaction model was augmented with a PKPD model for hypothetical antibiotics, for which the PK model was defined as a one-compartment distribution model with a distribution volume of 5 L and a 5-h half-life. Protein binding of the antibiotic was assumed to be absent. Antibiotic effects were implemented according to a PD model [25] in which the effect of the antibiotic on the bacterial growth rate was related to the drug concentration according to Eq. 7.

7 $$\begin{aligned} kg = kgrw - \left( \frac{(kgrw - kgmin) \cdot \frac{C_{AB}}{MIC}^H}{\frac{C_{AB}}{MIC}^H - \frac{kgmin}{kgrw}} \right) \end{aligned}$$

Here, *kg* equals the net growth rate of bacteria influenced by antibiotic treatment. *kgrw* is the net growth rate of bacteria in the absence of antibiotics. *kgmin* is the minimal bacterial growth rate at maximal antibiotic effect. *MIC* is the minimum inhibitory concentration which was set to 1 *μ *g/mL in these simulations. *H* is the hill coefficient of the sigmoidal concentration-effect relationship. In line with previous publications, bacteriostatic drugs are described with *kgmin* = -1 /h and bactericidal drugs with *kgmin* = -3 /h, time dependent drug effects are described with *H* = 0.5, and concentration dependent drug effects with *H* = 3 [26].

#### Literature-based parameter selection

To inform the parameter values of the mechanism-based model, a literature search was performed, using a hierarchical strategy. First, reported rate constants for phagocytosis, digestion, and bacterial induced cell death were searched. If these were not available, a second literature search was performed to retrieve experimental papers with *in vitro* data that allowed estimation of these parameter values. Inclusion criteria for the studies were (1) bacteria incubated with neutrophils or monocytes, where intracellular or extracellular bacterial concentrations (expressed as colony forming units (CFU) derived from measurements of total bacterial concentrations in a sample and measurements after washing and lysis of immune cells in the sample) were measured over time, or (2) natural apoptosis and phagocytosis-induced cell death of immune cells, with measurements of neutrophils or monocytes incubated with and without bacteria. The parameters were estimated by fitting the model equations to the relevant subset of data. The equations for phagocytosis and digestion (Eqs. 2 – 4) were fit to data meeting criterion 1, while the equation of apoptosis rate ($k_{i, apop}$) and phagocytosis-induced cell death rate ($k_{i,picd}$) (Eq. 5) was fitted to data meeting criteria 2. The extracted and estimated parameter values were incorporated in the full mechanism-based model of the innate immune response.

### Calibration

The full mechanism-based model of the innate immune response was calibrated to an *in vivo* setting by accounting for differences between *in vitro* and *in vivo* scenarios. *In vivo* immune cells, for instance, do not only degrade, as is the case *in vitro*, but also replicate and migrate, resulting in a relatively constant concentration over time. Therefore, we removed the apoptosis and pathogen-induced cell death processes of neutrophils and the empirical time-driven decay on monocyte activity. Additionally, bacterial growth may be lower *in vivo* compared to *in vitro* due to less optimal growth conditions, such as the availability of nutrients and local pH [27]. To identify the model parameters of the immune system that could be estimated based on *in vivo* data in the calibration, a global sensitivity analysis was performed to determine the sensitivity of the infection outcome to changes in parameter values. For this, the infection outcome was quantified as the area under the CFU versus time curve (AUC). Model parameters describing the immune system and bacterial growth rate were included in this sensitivity analysis, while *Bmax*, which is not expected to change with changes in immune cell concentrations or activity, was excluded from this analysis. The parameter values were varied within (patho)physiological ranges as described in Online Resource 1: Table S1. Two scenarios based on different host species were considered for the sensitivity analysis. The first scenario reflected *in vivo* studies in mice, in which immune cell concentrations and the bacterial inoculum were fixed to observed values in immune competent mice studies. The bacterial inoculum was $4\cdot 10^7 CFU/mouse$, with an average blood volume of 2.5 mL, this corresponded to $1.6\cdot 10^7 CFU/mL$. The second scenario reflected a clinical situation in which immune cell concentrations were fixed to values of an immune competent human. The bacterial inoculum was chosen to be in similar ranges as the *in vivo* study, and therefore set in this scenario to $10^6 CFU/mL$. To quantify the sensitivity of infection outcome to parameter values, the Sobol index [28] was used where the individual impact of a parameter on the outcome measure can be assessed by its first order index, and the impact of this parameter in combination with all its interactions with other parameters by its total order index. Parameters with higher first and/or total order indices have a higher impact towards the outcome measure.

Following the sensitivity analysis, a literature search was preformed to retrieve data to calibrate the model parameters to which the outcome variable were identified to be sensitive. The retrieved experimental data were obtained in immune competent mice, which were intravenously inoculated with live bacteria. The reported neutrophil and monocyte concentrations and the CFU counts that were determined within 24 h after inoculation were included. The parameters to which the infection outcomes were identified to be sensitive were fitted to these *in vivo* data, either by direct estimation of parameters or by estimation of a scaling factor as described in Online Resource 4.

### Evaluation of immune status on PKPD targets

The mechanism-based model, calibrated to *in vivo* murine data, was used to simulate dose fractionation studies to derive *in silico* PK/PD targets for patients with immune deficiencies. Calibration on patient data is not possible as the required PKPD data is unavailable and practically impossible to generate. Combinations of initial bacterial density and net growth rate were selected such that, in combination with one dose of the four types of antibiotics at t = 0 h, an infection was cleared within 24 h in simulated immune competent individuals, while increasing progression of infection was observed with increasing degree of immune deficiency. The simulations assumed a homogeneous bacterial population with an MIC of 1 *μ *g/mL, without resistance development over time (i.e. constant MIC). Simulations were performed to compare PK/PD targets between immune competent individuals (N: $5\cdot 10^6$ cells/mL, M: $0.6\cdot 10^6$ cells/mL), patients with monocytopenia (M: $0.2\cdot 10^6$ cells/mL), mild (N: $1.25\cdot 10^6$ cells/mL), moderate(N: $0.75\cdot 10^6$ cells/mL), and severe neutropenia (N: $0.5\cdot 10^6$ cells/mL), a combination of monocytopenia and severe neutropenia(N: $0.5\cdot 10^6$ cells/mL, M: $0.2\cdot 10^6$ cells/mL), and fully immune compromised patients (N: 0 cells/mL, M: 0 cells/mL) [29, 30]. In these simulations, total daily doses were divided over 1, 2, 3, or 6 doses in 24 h. PK/PD indices were calculated and fitted for each immune status and antibiotic type. The PK/PD target to achieve 2 log_10_ reduction in bacterial concentration (CFU/mL) after 24 h was determined with the fitted equation. The change in the PK/PD target for concentration dependent antibiotics (AUC/MIC) is expressed as fold change (FC) compared to the immune competent individual, while the change in PK/PD target for time dependent antibiotics is given as absolute change in % T >MIC compared to the immune competent individual. Furthermore, a comparison was also made in total dose required to achieve a 2 log_10_ reduction in bacterial density after 24 h between immune states. This comparison was drawn between doses given in one time, i.e. that have a dosing interval of 24 h and expressed as the FC compared to the immune competent individual.

### Software

Data were extracted from graphs using GetData Graph Digitizer (2.26.0.20). Nonlinear mixed-effects modeling and simulations were performed in R (v4.4.2) using nlmixr2 (v3.0.2) and rxode2 (v3.0.4). Visualization and model evaluation were carried out using the tidyverse (v2.0.0), scales (v1.3), patchwork (v1.3.0), kableExtra (v1.4.0), sensitivity (v1.30.1) and lhs (v1.2.0) packages.

**Table 1:** Overview of final parameter values

Process	Parameter	Abbreviation	Parameter	Unit	Ref.
			value		
Bacteria					
Kinetics	Net growth	$kgrw^a$	0.9	/h	[32]
	Maximal capacity	*Bmax*	$2.5 \cdot 10^{9}$	CFU/mL	[31]
Neutrophils					
Phagocytosis	Maximal phagocytic capacity	$k_{np,max}$	1.90	/h	[44]
	Concentration of neutrophils leading to 50% efficacy	$N_{p50}$	$6.92 \cdot 10^{5}$	cell/mL	[31]
	Ratio of bacteria to neutrophils leading to 50% efficacy	$B_N/N_{p50}$	3.92	CFU/cell	[31]
Digestion	Maximal digestive capacity	$k_{nd,max}$	5.61	/h	[31]
	Concentration of neutrophils leading to 50% efficacy	$$ N_{d50}$$	$$3.98 \cdot 10^{6}$$	cell/mL	[31]
	Ratio of bacteria to neutrophils leading to 50% efficacy	$B_N/N_{d50}$	2.84	CFU/cell	[31]
Monocytes					
Phagocytosis	Maximal phagocytic capacity	$k_{mp,max}$	1.77	/h	[44]
	Concentration of monocytes leading to 50% efficacy	$M_{p50}$	$2.73 \cdot 10^5$	cell/mL	[45–47]
	Ratio of bacteria to monocytes leading to 50% efficacy	$B_M/M_{p50}$	1.25	CFU/cell	[45–47]
	Hill factor	$\gamma _{p}$	5.54	-	[45–47]
Digestion	Maximal digestive capacity	$k_{md,max}$	6.23	/h	[45–47]
	Concentration of monocytes leading to 50% efficacy	$M_{d50}$	$3.98 \cdot 10^6$	cell/mL	[45–47]
	Ratio of bacteria to monocytes leading to 50% efficacy	$B_M/M_{d50}$	1.05	CFU/cell	[45–47]
	Hill factor	$\gamma _{d}$	14	-	[45–47]

$^{a}$ kgrw was calibrated to a clinically relevant value as described in result section *Evaluation of immune status on PK/PD targets*

**Fig. 1:**
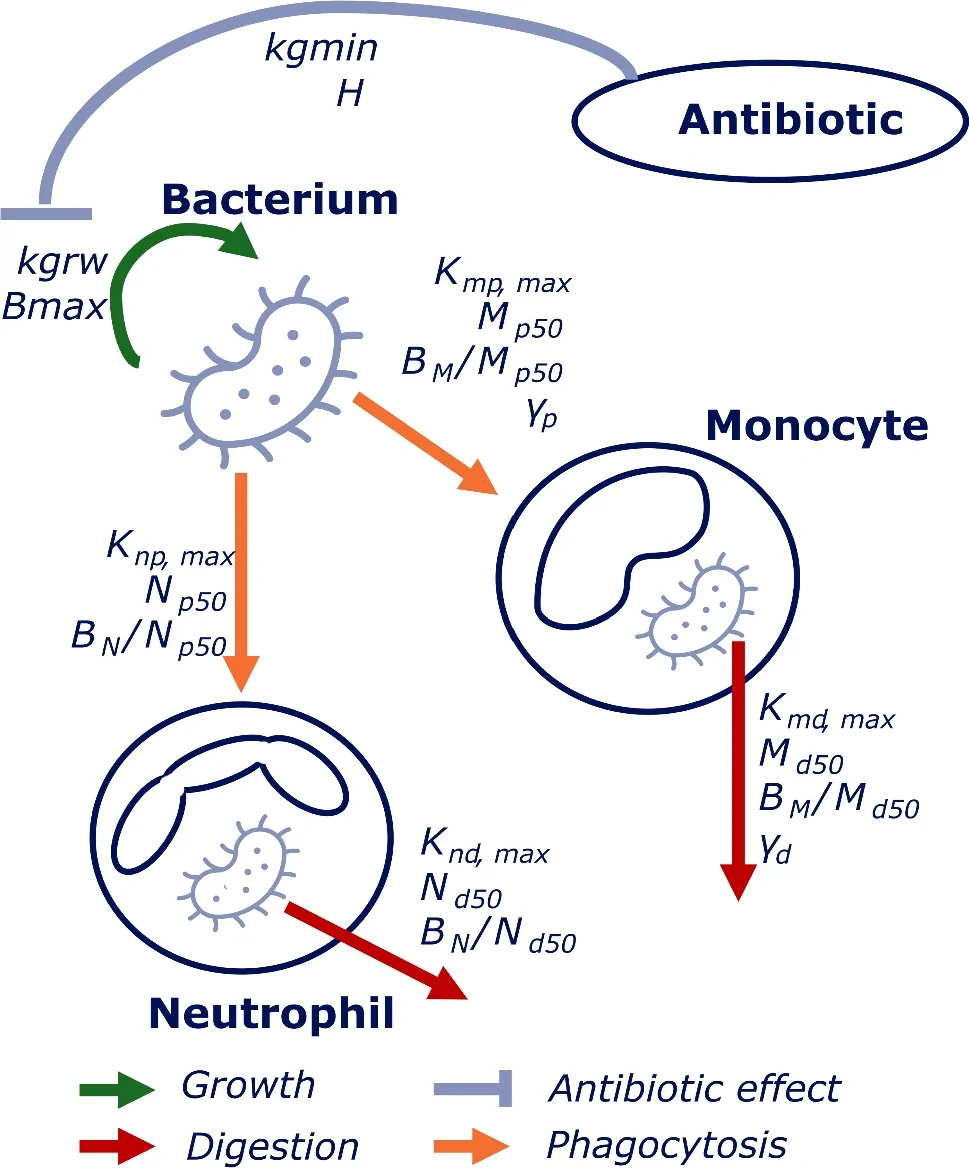
**Schematic of the mechanism-based model.** Bacterial net growth is indicated by the green arrow (Eq. 1). Bacteria are phagocytosed (orange arrows) by neutrophils and monocytes, both described by phagocytosis rates that are dependent on immune cell concentrations and capacity (Eqs. 8 and 11). The digestion (red arrows) of internalized bacteria is described in the same way (Eqs. 9 and 12). Finally, the antibiotic effect is shown by the light blue arrow (Eq. 7). The schematic shows the model structure after calibration

## Results

### Mechanism-based model development

The final model structure is depicted in Fig. 1, with the *in vitro* model parameter values (i.e., before calibration) being presented in Online Resource 1: Table S1, and the source of each value being presented in Online Resource 1: Table S2. The parameter descriptions and their units are presented in Table 1. Bacterial dynamics were described according to Eq. 1. Phagocytosis and digestion of bacteria by neutrophils were described with sigmoidal dependencies on neutrophil concentrations and capacity [31] (Eqs. 8 and 9). Additionally, neutrophil kinetics were described with apoptosis ($k_{n,apop}$) and phagocytosis-induced cell death ($k_{n,picd}$) rates that were estimated as described in the supplementary information (Online Resource 2) (Eq. 10). Phagocytosis-induced cell death was described as an on/off effect, where the presence of bacteria resulted in an additional death rate of neutrophils ($k_{n,picd}$), with an typical constant rate of 0.0092 /h. There was no relationship between the bacterial density and the death rate.

8 $$\begin{aligned} k_{np} = k_{np,max} \cdot \left( \frac{N}{N_{p50} + N} \right) \cdot \left( 1 - \frac{B_N/N}{B_N/N_{p50} + B_N/N} \right) \end{aligned}$$

9 $$\begin{aligned} k_{nd} = k_{nd,max} \cdot \left( \frac{N}{N_{d50} + N} \right) \cdot \left( 1 - \frac{B_N/N}{B_N/N_{d50} + B/N} \right) \end{aligned}$$

10 $$\begin{aligned} \frac{d}{dt}(N) = - k_{n,apop} \cdot N - k_{n,picd} \cdot N \end{aligned}$$

In these equations, $k_{np}$ and $k_{nd}$ are phagocytosis and digestion rate constants of neutrophils, $k_{np,max}$ and $k_{nd,max}$ are the maximum rate constants. $N_{p50}$ and $N_{d50}$ are the neutrophil concentrations leading to half the maximum phagocytosis and digestion rates. $B_N/N_{p50}$ and $B_N/N_{d50}$ are the ratios between intracellular bacteria and neutrophil concentrations leading to half the maximum phagocytosis and digestion rates.

Phagocytosis and digestion of bacteria by monocytes were also described with sigmoidal dependencies on monocyte concentration and capacity (Eqs. 11 and 12). The parameters were estimated on the collected *in vitro* data, with the exception for $M_{d50}$ for which no data was available. $M_{d50}$ was therefore assumed to be equal to the corresponding value for the neutrophils ($N_{d50}$), as both immune cells have similar mechanisms of digestion. No data was retrieved that allowed for the estimation of death rates of monocytes, therefore, a time-driven decay of both the phagocytosis and digestion rates of monocytes was estimated. The estimation of the phagocytosis and digestion rates of monocytes is described in the supplementary information (Online Resource 3).

11 $$\begin{aligned} k_{mp} &= k_{mp,max} \cdot \left( \frac{M}{M_{p50} + M} \right) \\& \cdot \left( 1 - \frac{(B_M/M) ^{ \gamma _p}}{(B_M/M_{p50})^{ \gamma _p} + (B/M)^{ \gamma _p}} \right) \cdot e^{-k_{decay,mp} \cdot t} \end{aligned}$$

12 $$\begin{aligned} k_{md}& = k_{md,max} \cdot \left( \frac{M}{M_{d50} + M} \right) \\&\cdot \left( 1 - \frac{(B_M/M) ^ { \gamma _d}}{(B_M/M_{d50})^{ \gamma _d} + (B/M)^{ \gamma _d}} \right) \cdot e^{-k_{decay,md} \cdot t} \end{aligned}$$

In these equations, $k_{mp}$ and $k_{md}$ are phagocytosis and digestion rate constants of monocytes, $k_{mp,max}$ and $k_{md,max}$ are the maximum rate constants. $M_{p50}$ and $M_{d50}$ are the monocytes concentrations leading to half the maximum phagocytosis and digestion rates. $B_M/M_{p50}$ and $B_M/M_{d50}$ are the ratios between intracellular bacteria and monocyte concentrations leading to half the maximum phagocytosis and digestion rates. $k_{decay,mp}$ and $k_{decay,md}$ are the decay rate constants, and *t* is time.

**Fig. 2:**
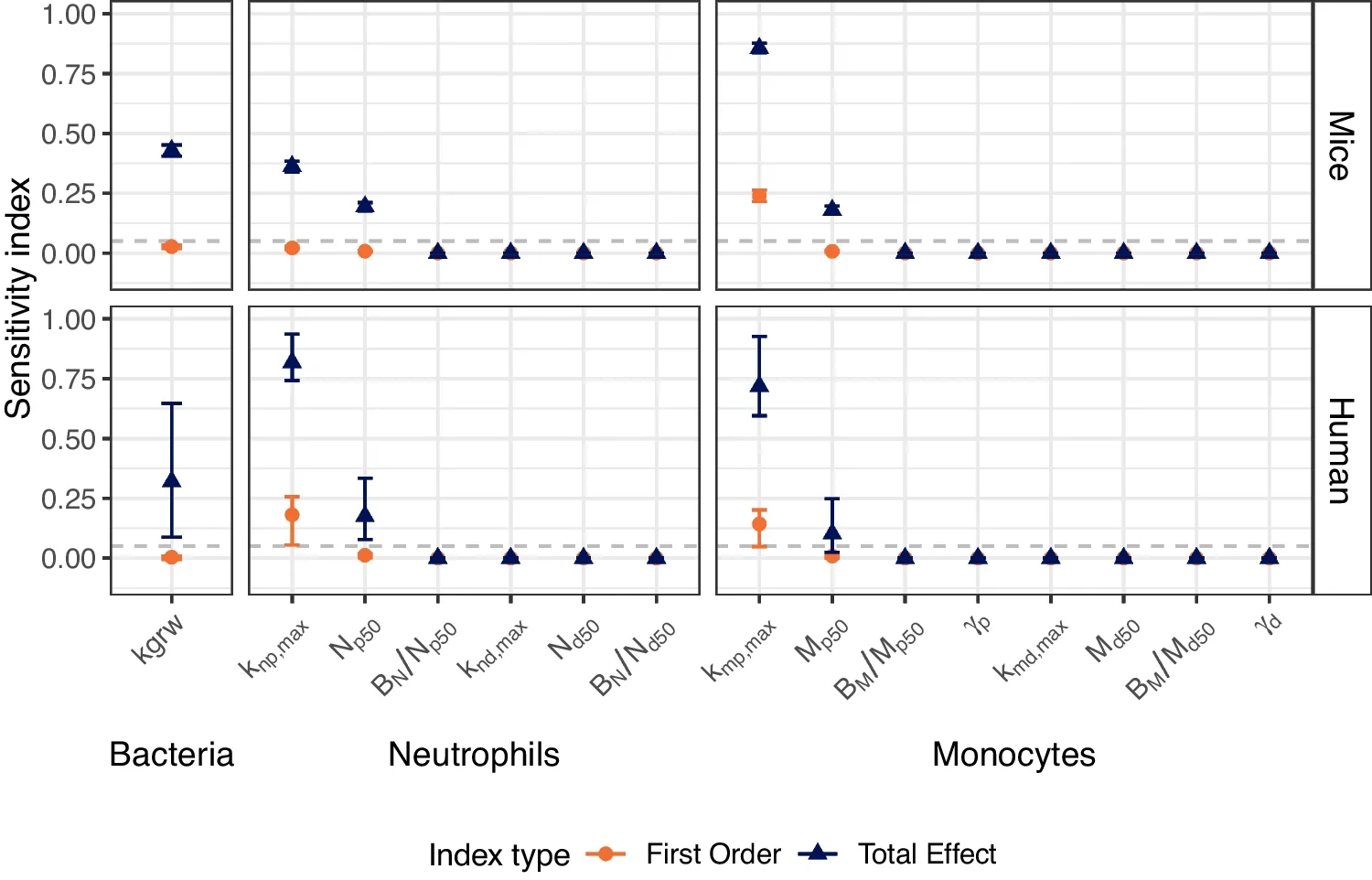
**First order sensitivity indices (orange circle) and total order indices (blue triangle) with 95% confidence interval for the scenarios of immune competent mice and immune competent humans.** The first order index shows the individual impact of a parameter on the outcome measure, the AUC of the bacterial density (CFU/mL) over time. The impact of this parameter in combination with all its interactions with other parameters is shown by its total order index. The dashed line at 0.05 was included to indicate parameters contributing at least 5% to the variance in the model output, which was considered a threshold for practical relevance

### Calibration

To identify model parameters that could be fitted to *in vivo* data, to calibrate the model to represent *in vivo* conditions, a general sensitivity analysis was performed for two scenarios. Before the sensitivity analysis, the bacterial growth rate, *kgrw*, was lowered from 1.451 /h to 0.5885 /h to better represent *in vivo* conditions [32]. The sensitivity of the parameters was quantified by the Sobol index, where parameters with higher first and/or total order indices have a higher impact towards the outcome measure (Fig. 2). In both scenarios, the maximum phagocytosis rate of monocytes ($k_{mp,max}$) was found to impact the AUC of bacterial CFU over time, with a first order index of 0.24 and 0.14 for the mice and human, respectively. The maximum phagocytosis rate of neutrophils ($k_{np,max}$) and the growth rate of bacteria (*kgrw*) were also found to be impactful. With the higher neutrophil concentration in humans compared to mice, changes in the maximum phagocytosis rate of neutrophils ($k_{np,max}$) impacted the predicted AUC of bacterial CFU more for humans compared to mice (first order index of 0.18 vs. 0.022, respectively). CFU over time was also found to be sensitive to changes in the values of the half maximum ratio of bacteria to neutrophils of phagocytosis ($B_N/N_{p50}$) and the half maximum monocyte concentration of phagocytosis ($M_{P50}$). Furthermore, the five parameters showed strong interactions with each other, showed by increase in sensitivity index for total effect, underscoring the interdependence of different components of the immune response. As not all five parameters could be estimated on the available data and as our focus was on describing the bacterial elimination by phagocytosing cells, the two maximum rates of phagocytosis, $k_{mp,max}$ and $k_{np,max}$, were selected to be estimated based on *in vivo* data (Online Resource 1: Table S3). In the available data, bacterial concentrations were measured in four mice and these data were used to estimate the two maximum phagocytosis rates as described in Online Resource 4. Given the limited *in vivo* data, one scaling factor was estimated for both parameters, which showed the clinical values to be 77% lower than the *in vitro* values. The final model parameter values are shown in Table 1.

**Fig. 3:**
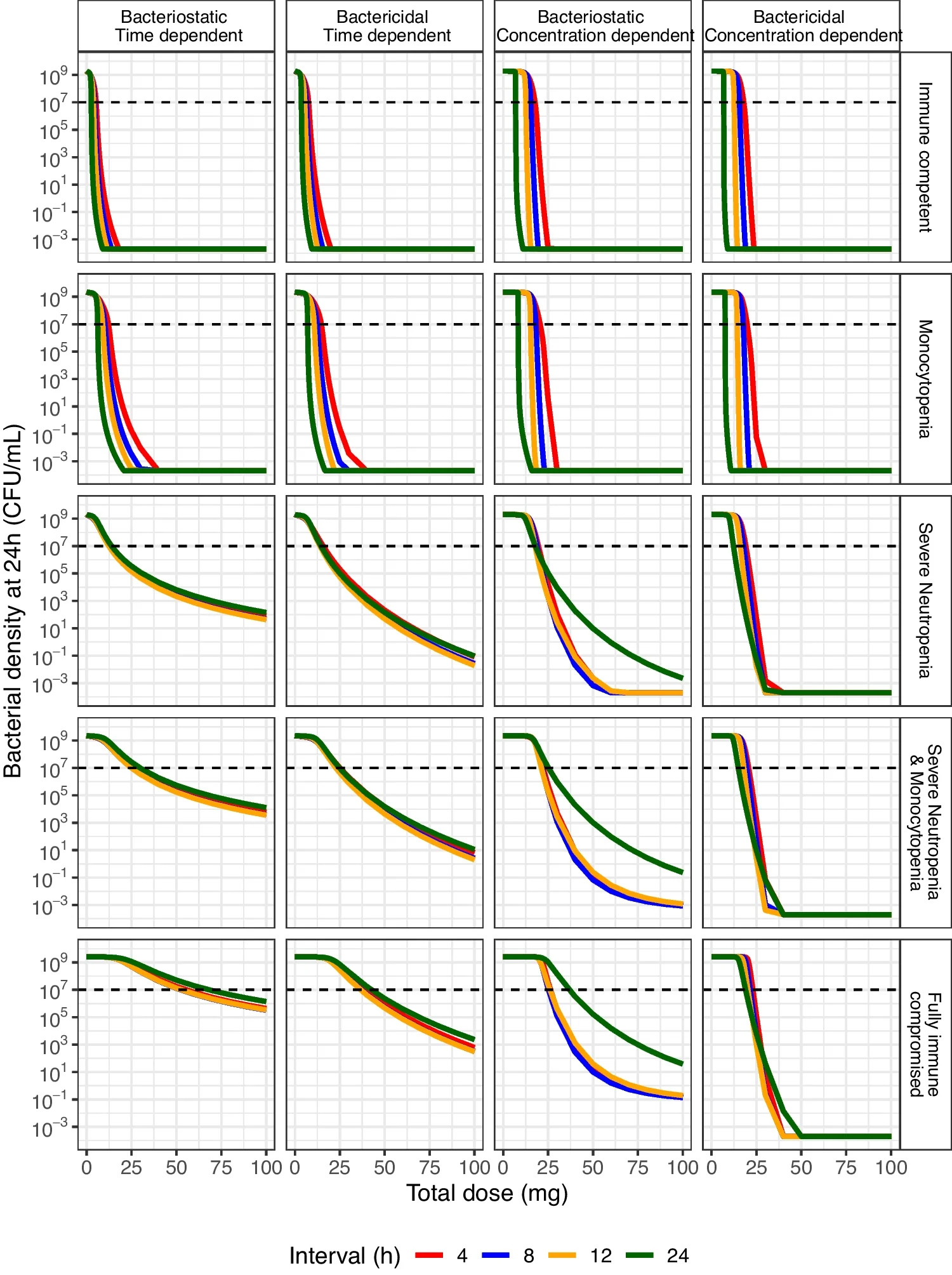
**Dose fractionation simulations for a subset of five immune states and four antibiotic types.** Shown is the predicted bacterial density (CFU/mL) at 24 h over a range of total doses, given in either 1 dose (interval of 24 h, green), in 2 doses (interval of 12 h, yellow), in 3 doses (interval of 8 h, blue), or in 6 doses (interval of 4 h, red). Indicated with the dotted horizontal line is a 2 log_10_ drop in bacterial density

**Fig. 4:**
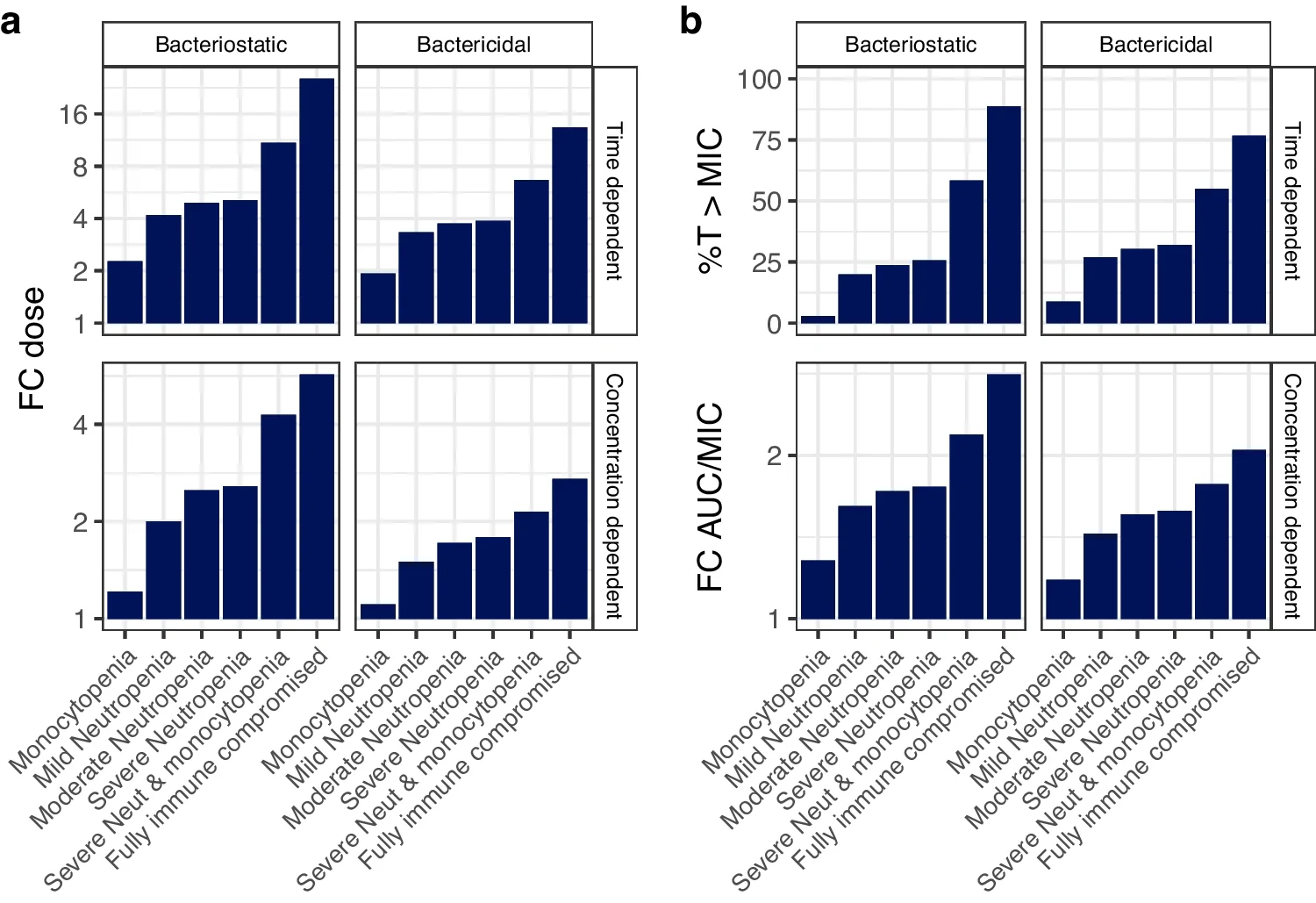
**Summarized results of the dose fractionation studies for six immune states for the four types of antibiotics.** The doses included in panel a are all administered as a single dose at T=0. Shown are both the fold change in dose (a) for the four types of antibiotics and the PK/PD target changes (b) for time dependent antibiotics as the absolute change in %T>MIC compared to the immune competent individual; and for the concentration dependent antibiotics as the log_2_ fold change in AUC/MIC compared to the immune competent individual. FC = log_2_ fold change, MIC = minimum inhibitory concentration, T>MIC = time above MIC, AUC = area under the curve

### Evaluation of immune status effect on PK/PD targets

An initial bacterial density of $10^9 CFU/mL$ and a growth rate of 0.9/*h* were selected as described in Online Resource 1: Fig. S1. The dose fractionation simulations showed increasing doses required to achieve the 2 log_10_ reduction in bacterial concentration (CFU/mL) after 24 h with worsening immune deficiency (Fig. 3, Online Resource 1: Fig. S2). The fitted PK/PD indices also show the increased dose requirements (Online Resource 1: Fig. S3). With the same total dose, the impact of different dosing intervals is limited, with the exception of 24 h dosing interval for bacteriostatic concentration dependent antibiotics. At this regimen, the antibiotic concentration decreased too quickly to a concentration that is too low to exert bacteriostasis, allowing bacterial growth toward the end of the dosing interval and resulting in a higher bacterial density at 24 h than with the other dosing intervals (Online Resource 1: Fig. S4). In contrast, the applied regimen for the bactericidal antibiotics substantially reduced the bacterial population early in the dosing interval, leaving insufficient viable bacteria for notable regrowth at times when antibiotic concentrations declined.

The comparison between total dose, given as one dose in 24 h, and PK/PD targets between immune states are visualized in Fig. 4. This figure indicates how much the dose increases for increasing degrees of immune deficiency for all antibiotic types (Fig. 4a). The fold change in dose is up to five times as high for the time dependent antibiotics compared to the concentration dependent antibiotics, up to a fold change of 25.5 compared to 5.7 for bacteriostatic time and concentration dependent antibiotics, respectively, in fully immune compromised patients. The PK/PD targets also show increasing patterns along with increasing degrees of immune deficiency (Fig. 4b). In an immune competent individual, an antibiotic dose leading to concentrations below the MIC is sufficient to achieve a 2 log_10_ reduction in CFU, thereby resulting in a PK/PD target of 0% T>MIC for time dependent antibiotics, to reach the desired kill within 24 h. However, in case of severe neutropenia combined with monocytopenia, an increase to 55% and 58% T>MIC is needed for bactericidal and bacteriostatic antibiotics, respectively. For the same immune deficiency, in comparison to the immune competent individual, a 1.7 and 2.2 times higher AUC/MIC has to be reached for sufficient kill within 24 h for bactericidal and bacteriostatic drugs, respectively.

## Discussion

A mechanism-based model was developed for the early immune-mediated bacterial clearance by neutrophils and monocytes during bacteremia to quantify how immune impairment alters antibiotic PK/PD targets. Our simulations illustrate how much an increasing degree of immune deficiency is associated with higher PK/PD targets to achieve comparable pharmacodynamic outcomes, underscoring the role of host immunity in determining effective antibiotic dosing regimen. Previous mathematical models have described host–pathogen interactions in specific infections, mostly pneumococcal pneumonia, often incorporating bacterial dynamics together with immune-cell activation, cytokine responses, tissue injury, or barrier function [15–20, 33]. Other models have investigated the combined contribution of generic antibiotics and host immunity to bacterial clearance and treatment outcomes [22, 23]. The present study builds on these approaches by linking mechanistic phagocyte-mediated bacterial clearance to conventional antibiotic PK/PD indices and by quantifying how graded deficiencies in specific immune-cell populations affect the targets required to achieve a predefined pharmacodynamic response. Our study therefore provides quantitative insight into the impact of host immunity on PK/PD targets, which hitherto have remained mostly qualitative or anecdotal.

The model focused on phagocytosis and digestion as key mechanisms of bacterial elimination and included the dominant phagocytic innate immune cells in early bacteremia: neutrophils and monocytes [21]. Both the bacteria and antibiotic were implemented as generic elements with adjustable parameters, enabling exploration of bacteria characteristics and drug classes. Pharmacodynamic parameters were used to capture time- versus concentration-dependent behavior, as well as bacteriostatic versus bactericidal activity. The processes included in the model reflect the acute immune response and model-based predictions of clinical outcomes should therefore be limited to the first 24 h after infection.

Given that the *in vivo* experiments used in this study provided only a single 24 h CFU measurement, not all model parameters could be estimated from the *in vivo* data alone. We, therefore, estimated only the parameters to which bacterial CFU was most sensitive using the *in vivo* data, while fixing the remaining parameters to the *in vitro* values to leverage all available information in the best possible manner. Because *in vitro* conditions facilitate frequent bacteria–immune cell interactions, the hypothesis was that the effective interaction rates would be lower *in vivo*, consistent with observations that pathogens may evade immune responses by localizing within tissues [31]. Model calibration supported this hypothesis, requiring a 77% reduction in neutrophil and monocyte phagocytosis rates to reproduce *in vivo* mice dynamics. The sequential use of *in vitro* and *in vivo* evidence provides mechanistic insight into how the immune response contributes to early bacterial clearance.

Using the calibrated model across immune states, we found that increasing degrees of immune deficiency were associated with higher PK/PD targets, consistent with clinical observations [7]. In translational modeling, *in vitro*-to-*in vivo* extrapolation often relies on immune compromised animal data and may implicitly assume a negligible immune contribution [34]. In contrast, our model predicts meaningful differences in PK/PD targets when immune mediated clearance is present versus when immunity is absent, highlighting the importance of incorporating immune dynamics when evaluating antibiotic dosing strategies. This suggests that widely used targets derived from immune compromised animals may function as low-immunity benchmark, rather than a universal requirement across hosts. In addition to this host-dependent shift, antibiotic-class-dependent differences were also observed. The shifts in PK/PD targets were more pronounced for time-dependent antibiotics than for concentration-dependent antibiotics. One possible explanation is that the prolonged exposure required for time-dependent antibiotics increases reliance on immune-mediated clearance, making target increases more apparent as immune function declines.

The developed mechanism-based model serves as a base model of the innate immune response, designed to evaluate the impact of immune cell deficiencies of the main circulating phagocytic cell populations on PK/PD targets. Neutrophils and monocytes were selected because they play a central role in the early clearance of bacteria and together account for up to 70% of the circulating immune cells [21, 35]. By representing these two cell populations, the model captures a substantial proportion of the cellular innate immune response present during the first 24 h of infection. While neutrophils are commonly included in models of the innate immune response, monocytes are less frequently represented, likely because they are often considered primarily as precursors of monocyte-derived macrophages following recruitment to infected tissues [20, 33, 36]. Two previous studies explicitly described monocytes and their contribution to cytokine production, but neither incorporated monocyte-mediated bacterial phagocytosis and intracellular digestion [33, 37]. Consistent with the objectives of this study and existing innate immune modeling approaches, interactions with other immune components, such as cytokine-mediated regulation, were not represented [16, 17]. Instead of simplifying the immune response by grouping pro- and anti-inflammatory responses[38], or innate and adaptive immune responses [22], we chose to specifically describe neutrophils and monocytes, as main drivers of the innate immune response. Nevertheless, incorporating cytokine-mediated regulation and other immune cell dynamics may be relevant extensions of the current model. The simulations also assumed fixed numbers of neutrophils and monocytes; allowing immune cell concentrations and phagocytic activity to vary over time could better reflect the *in vivo* setting and improve predictions of bacterial elimination, particularly beyond the 24 h window considered in this study.

To generate informative dynamics within the 24 h acute window, a high initial bacterial density ($10^9 CFU/mL$) was required. Although human bacteremia is generally associated with a bacterial load below 100 CFU/mL [39], our simulations showed that in most cases the immune system is capable of clearing infections at this inoculum, without the need for antibiotic treatments. We, therefore, consider the relatively low bacterial load observed in the clinical context to represent the bacteria in the systemic circulation that escape the immune system when the immune system is saturated or depleted by a continuous influx of bacteria from a peripheral infection site. As our model does not include a compartment for bacterial transfer from an infection site into the bloodstream, the inoculum in our simulations should be interpreted as an effective “challenge burden” that is a proxy for the saturation of the immune system by continuous bacterial seeding rather than a literal bloodstream concentration. This inoculum, selected based on simulations, resulted in a bacterial concentration that substantially exceeded the concentrations of circulating immune cells, reflecting a stage at which the phagocytic capacity of the immune system is saturated or depleted, allowing bacteria to persist in the bloodstream and necessitating antibiotic treatment. Although our model simulates an immediate bacterial challenge, whereas continuous bacterial seeding may evolve over a longer time-frame that extends beyond the 24 h window considered in this study, the selected inoculum resulted in patterns of bacterial growth and eradication by the immune system that are in line with clinically relevant patterns for a wide range of immune deficiencies (Online Resource 1: Fig. S1). Due to the discrepancies in simulated and clinically observed bacterial densities, the emphasis of our work is on *relative* PK/PD target shifts across immune states rather than on absolute targets as direct clinical thresholds.

Clinical application of this approach requires further translational steps, particularly because the current model uses a generic pathogen and antibiotic. To support patient-level dosing decisions, pathogen-specific dynamics, drug-specific PK, and patient-specific immune characteristics would need to be incorporated. In addition, altered antibiotic PK due to disease-related changes in physiology, organ function, and inflammatory status may further impact required antibiotic dose adjustments [40, 41]. Pharmacodynamic factors such as resistance development during treatment, partly driven by the frequent use of broad-spectrum antibiotics and high transmission rates in ICU settings, may also influence effective targets [42]. Extending the bacterial model component to include both antibiotic-sensitive and -resistant phenotypes could improve generalizability [43]. Incorporating these complexities may enhance clinical relevance and predictive performance, and could further modulate the PK/PD target shifts observed in our simulations relative to immune competent individuals.

In conclusion, our mechanism-based model quantitatively describes the impact of neutrophil and monocyte suppression on PK/PD targets during bacteremia. These findings support approaches to account for host immune status when evaluating dosing strategies, particularly in immune compromised patients.

## Supplementary Information

Below is the link to the electronic supplementary material.

Supplementary file 1 (pdf 629 KB)

Supplementary file 2 (pdf 96 KB)

Supplementary file 3 (pdf 196 KB)

Supplementary file 4 (pdf 45 KB)

## Data Availability

Data is available upon request

## Code Availability

Available upon request.
