# Supplementary material for: A mechanism-based model of immune status effects on antibiotic PK/PD targets in bacteremia
Source: J Pharmacokinet Pharmacodyn. 2026 Sep 22;53(6):53. doi: 10.1007/s10928-026-10063-6 (PMC13597578; doi:10.1007/s10928-026-10063-6)
Supplement: Supplementary file 1 [file 10928_2026_10063_MOESM1_ESM.pdf]

A mechanism-based model of immune status effects on antibiotic PK/PD targets in bacteremia

Marinda van de Kreeke[1†], Anh Duc Pham[1†], Michelle Mehciz[1], Tamara Jordens[1], Marnix G. Uyterlinde[1], Rob C. van Wijk[1], Linda B.S. Aulin[1], Catherijne A.J. Knibbe[1,2], Elke H.J. Krekels[1,3], Laura B. Zwep[1], J.G. Coen van Hasselt[1*]

[1]Division of Systems Pharmacology and Pharmacy, Leiden Academic Centre for Drug Research, Leiden, The Netherlands
[2]Department of Clinical Pharmacy, St. Antonius Hospital, Nieuwegein, The Netherlands
[3]Certara Inc, Radnor, PA, USA

[*]Corresponding author. E-mail: coen.vanhasselt@lacdr.leidenuniv.nl
†These authors contributed equally to this work.

Journal of Pharmacokinetics and Pharmacodynamics

Table S1 ***In vitro*** **parameter values and parameter ranges used in the global sensitivity analysis.** Parameter ranges are based on physiological and pathophysiological ranges, if these could be retrieved from literature. Otherwise, a range of 50 - 200% was taken

**Process**	**Parameter**	**Abbreviation**	***In vitro*** **parameter value [source]**	**Range in sensitivity analysis [source]**	**Unit**
**Bacteria**					
Kinetics	Net growth	$kgrw$	1.451 [1]	0.277 – 0.90 [2]	/h
	Maximal capacity	$Bmax^a$	$2.5 \cdot 10^9$ [3]	-	CFU/mL
**Neutrophils**					
Kinetics	Natural death rate	$k^a_{n,apop}$	0.0371 [4–15]	-	/h
	Bacterial induced death rate	$k^a_{n,bid}$	0.0092 [4–15]	-	/h
Phagocytosis	Maximal phagocytic capacity	$k_{np,max}$	8.35 [3]	0.835 – 16.7 [3, 16–18]	/h
	Concentration of neutrophils leading to 50% efficacy	N_{p50}	$6.92 \cdot 10^5$ [3]	$5 \cdot 10^5 - 7.7 \cdot 10^6$ [19]	cell/mL
	Ratio of bacteria to neutrophils leading to 50% efficacy	B_N/N_{p50}	3.92 [3]	0.5 – 148 [20, 21]	CFU/cell
Digestion	Maximal digestive capacity	$k_{nd,max}$	5.61 [3]	0.561 – 11.22 [3, 16–18]	/h
	Concentration of neutrophils leading to 50% efficacy	N_{d50}	$3.98 \cdot 10^6$ [3]	$5 \cdot 10^5 - 7.7 \cdot 10^6$ [19]	cell/mL
	Ratio of bacteria to neutrophils leading to 50% efficacy	B_N/N_{d50}	2.84 [3]	0.5 – 148 [20, 21]	CFU/cell
**Monocytes**					
Phagocytosis	Maximal phagocytic capacity	$k_{mp,max}$	7.78 [22–24]	0.778 – 15.56 [3, 16–18, 25]	/h
	Concentration of monocytes leading to 50% efficacy	M_{p50}	$2.73 \cdot 10^5$ [22–24]	$2 \cdot 10^5 - 1 \cdot 10^6$ [26]	cell/mL
	Ratio of bacteria to monocytes leading to 50% efficacy	B_M/M_{p50}	1.25 [22–24]	0.5 – 116 [22]	CFU/cell
	Hill factor	γ_p	5.54 [22–24]	2.77 – 11.08	-
	Decay of efficacy	$k^a_{decay,mp}$	0.657 [22–24]	-	/h
Digestion	Maximal phagocytic capacity	$k_{md,max}$	6.23 [22–24]	0.623 – 12.46 [3, 16–18, 25]	/h
	Concentration of monocytes leading to 50% efficacy	M_{d50}	$3.98 \cdot 10^6$ [22–24]	$2 \cdot 10^5 - 1 \cdot 10^6$ [26]	cell/mL
	Ratio of bacteria to monocytes leading to 50% efficacy	B_M/M_{d50}	1.05 [22–24]	0.5 – 116 [22]	CFU/cell
	Hill factor	γ_d	14 [22–24]	7 – 28	-
	Decay of efficacy	$k^a_{decay,md}$	1.1 [22–24]	-	/h

[a] Parameter excluded during sensitivity analysis

Table S2 Overview of obtained literature used for *in vitro* parameter selection, including studies from which parameters were directly extracted and *in vitro* data from which parameter values were estimated

Information extracted	Reference	Bacteria	Inoculum	Cell concentration	Measurement
Parameter values	[1]	*E. coli*, *P. aeruginosa*, *S. enterica*, *S. aureus*, *V. cholerae*	-	-	-
	[3]	*S. epidermidis*, *S. aureus*, *E. coli*, *S. pyogenes*, *Corynebacterium*	-	-	-
Neutrophil concentrations *in vitro*	[5]	-	-	$5 \cdot 10^6$ N/mL	Morphological assessment
	[6]	-	-	10^6 N/mL	Annexin V-PI staining
	[4]	*E. coli*	$2.5 \cdot 10^7$ CFU/mL	10^6 N/mL	Hypo-diploid DNA
	[7]	*M. tuberculosis*	$4 \cdot 10^7$ CFU/mL	$2 \cdot 10^6$ N/mL	Flow cytometry
	[8]	*S. pneumoniae*	$1.5 \cdot 10^8$ CFU/mL	$5 \cdot 10^6$ N/mL	Trypan blue
	[9]	*A. phagocytophilum*	$2.5 \cdot 10^7$ CFU/mL	$2 \cdot 10^6$ N/mL	Morphological assessment
	[10]	*A. phagocytophilum*	10^8 CFU/mL	$3 \cdot 10^6$ N/mL	Morphological assessment
	[11]	*B. cenocepacia*	$3 \cdot 10^8$ CFU/mL	10^7 N/mL	Annexin V and EthD
	[12]	*C. pneumoniae*	10^7 CFU/mL	10^7 N/mL	Morphological assessment
	[13]	*S. aureus*	10^7 CFU/mL	10^6 N/mL	PI stained, fragmented DNA
	[14]	*B. hermsii*, *S. aureus*, *S. pyogenes*, *L. monocytogens*, *B. cepacia*	$3 \cdot 10^6$ CFU/mL	$3 \cdot 10^5$ N/mL	TUNEL assay
	[15]	*S. aureus*	$0.3, 1, 3 \cdot 10^7$ CFU/mL	10^6 N/mL	PI and Annexin V staining
Bacteria concentrations *in vitro*	[22]	*S. aureus*, *E. coli*	$5 \cdot 10^3 - 5 \cdot 10^9$ CFU/mL	$5 \cdot 10^4 - 5 \cdot 10^6$ M/mL	Intracellular & extracellular
	[23]	*S. aureus*, *E. coli*	$4 \cdot 10^7$ CFU/mL	$2 \cdot 10^6$ M/mL	Intracellular & extracellular
	[24]	*E. coli*, *S. pyogenes*	$2.5 \cdot 10^7$ CFU/mL	$2.5 \cdot 10^6$ M/mL	Intracellular

Table S3 Overview of obtained literature used for *in vivo* calibration

Information extracted	Reference	Bacteria	Inoculum	Cell concentration	Animal model
Parameter values	[2]	*E. coli*, *S. aureus*	-	-	Sepsis mouse
Bacteria concentrations *in vivo*	[27]	*E. coli*	$4 \cdot 10^7$ CFU/mL	$1.7 \cdot 10^5$ N/mL, $1.4 \cdot 10^6$ M/mL	Sepsis mouse

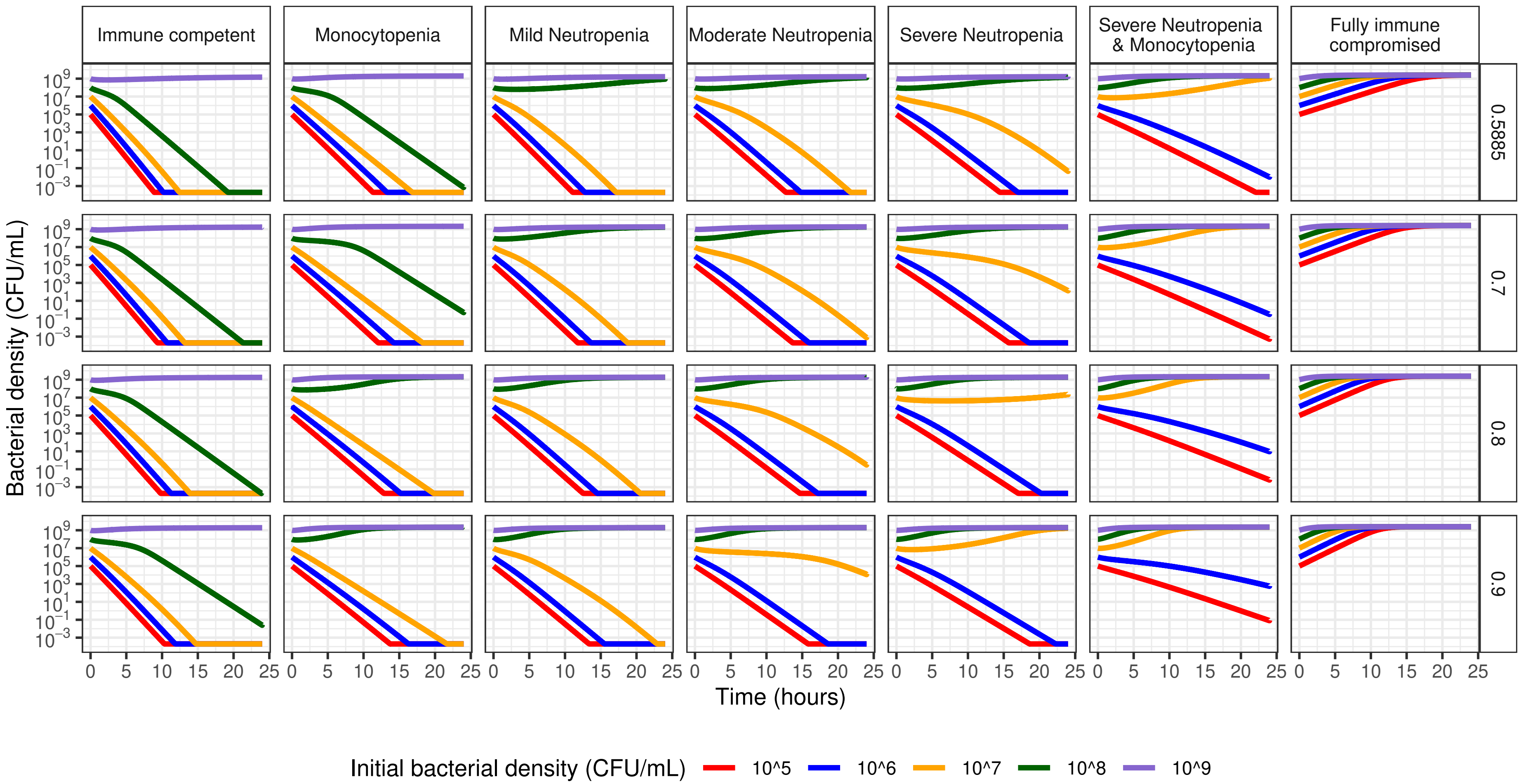

Fig. S1 Selection of initial bacterial density and $kgrw$ before the dose fractionation simulations. Shown is the predicted bacterial density (CFU/mL) over time, for seven immune states (columns), four different $kgrw$ values (rows) and five initial bacterial densities (colored lines). Combinations of initial bacterial density and $kgrw$ were selected such that an infection was cleared within 24 hours in simulated immune competent individuals only when one antibiotic dose was added, while increasing progression of infection was observed with increasing degree of immune deficiency. An initial bacterial density of $10^9 CFU/mL$ and $kgrw$ of $0.9/h$ were selected. $kgrw$ = net bacterial growth rate

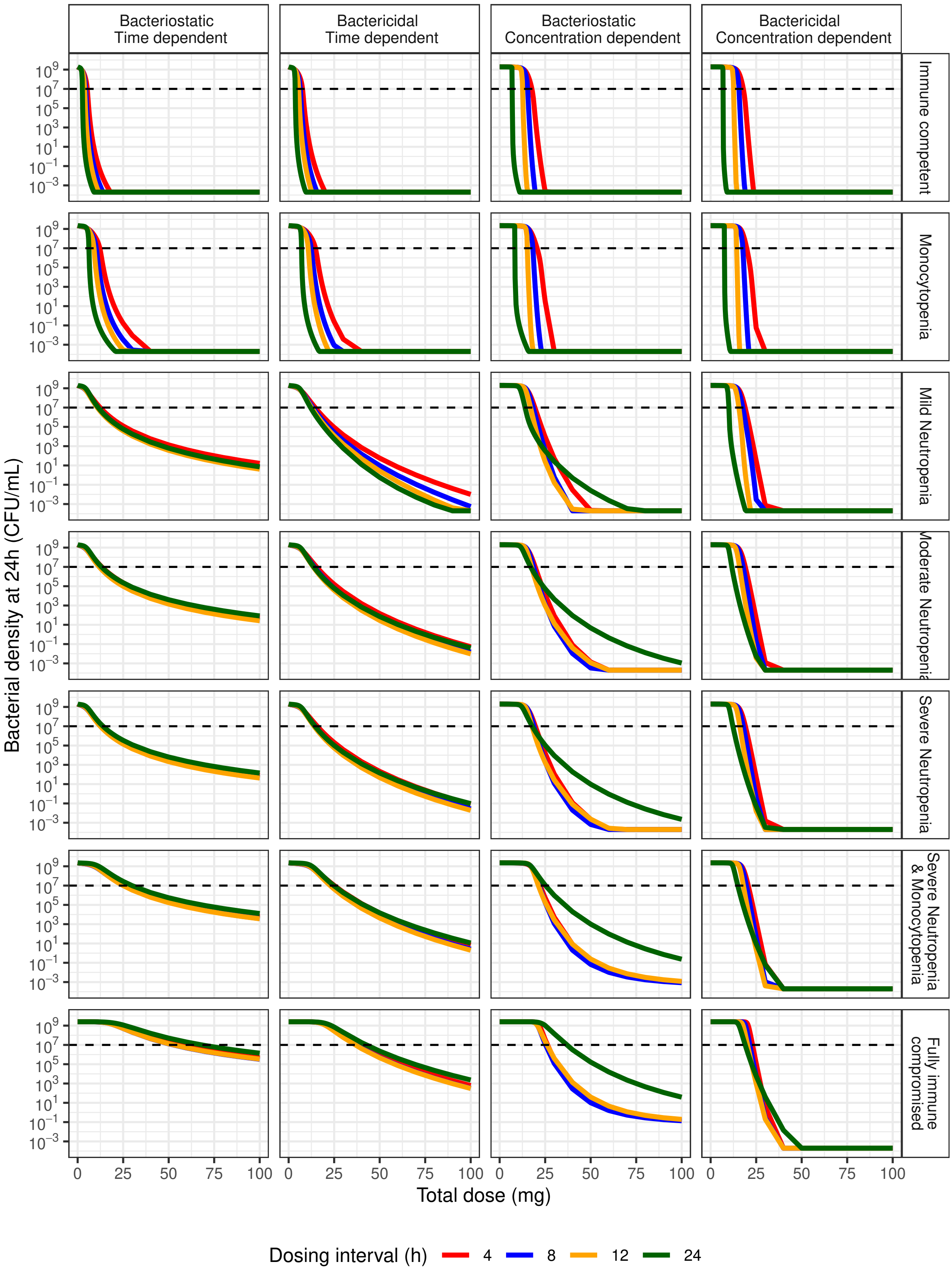

Fig. S2 Simulations of complete dose fractionation studies for seven immune states and four antibiotic types. In addition to Fig. 3 in the main text, are the mild and moderate neutropenia included here as well. Shown is the predicted bacterial density (CFU/mL) at 24 hours over a range of total doses, given in either 1 dose (interval of 24 hours, green), in 2 doses (interval of 12 hours, yellow), in 3 doses (interval of 8 hours, blue), or in 6 doses (interval of 4 hours, red). Indicated with the dotted horizontal line is a 2 $\log_{10}$ drop in bacterial density

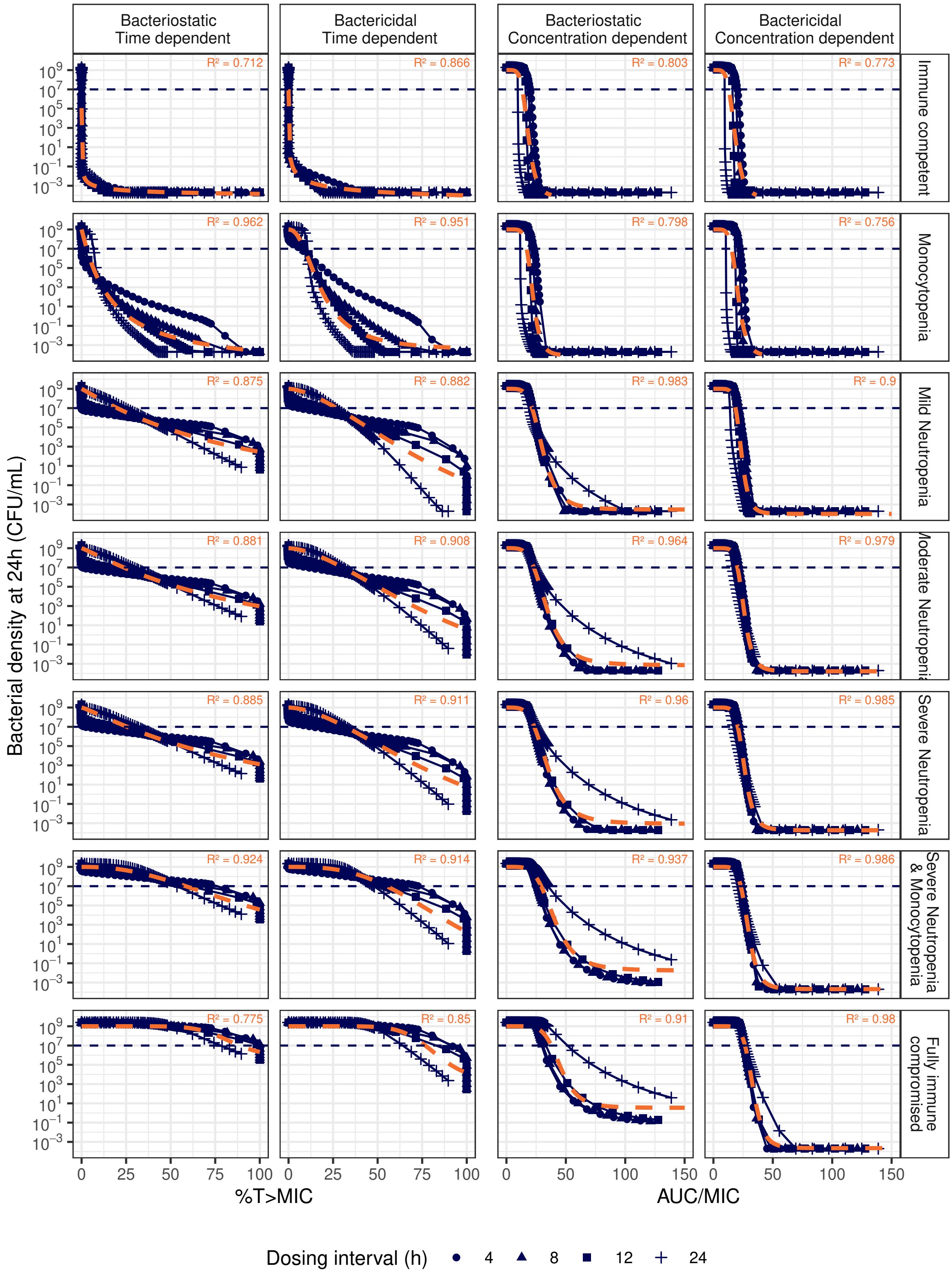

Fig. S3 Fitted PK/PD indices for seven immune states. Shown is the predicted bacterial density (CFU/mL) at 24 hours over a range of PK/PD indices, given in either 1 dose (interval of 24 hours, plus), in 2 doses (interval of 12 hours, square), in 3 doses (interval of 8 hours, triangle), or in 6 doses (interval of 4 hours, circle). Indicated with the dotted horizontal line is a 2 $\log_{10}$ drop in bacterial density. The dashed orange line shows the fitted relationship between bacterial density and PK/PD index, along with the R-squared value in the top right corner of each facet. The four antibiotic types are grouped based on their PK/PD index: %T>MIC for time-dependent antibiotics, AUC/MIC for concentration-dependent antibiotics. MIC = minimum inhibitory concentration, T>MIC = time above MIC, AUC = area under the curve

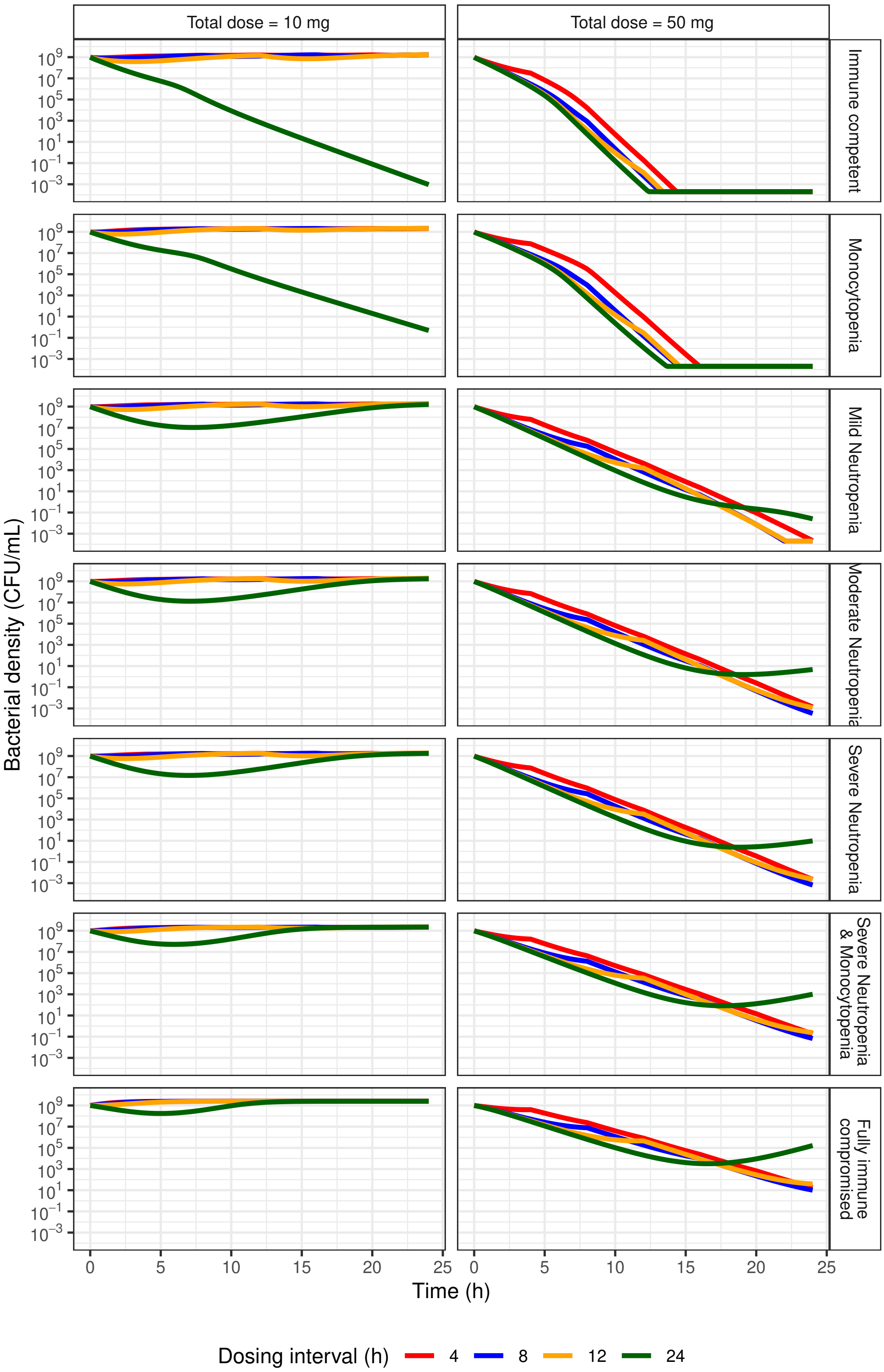

Fig. S4 Simulation of the bacterial density over time for two total doses for a bacteriostatic - concentration dependent antibiotic. Two total doses of 10 mg and 50 mg are compared for the bacteriostatic - concentration dependent antibiotic across seven immune states (rows) and four dosing intervals (colors)

References

[1] B. Gibson et al. "The distribution of bacterial doubling times in the wild". en. In: *Proc R Soc B* 285.1880 (June 2018). DOI: 10.1098/rspb.2018.0789.

[2] A. Dalhoff. "Differences between bacteria grown in vitro and in vivo". en. In: *J Antimicrob Chemother* 15.suppl A (Jan. 1985), pp. 175–195. DOI: 10.1093/jac/15.suppl_A.175.

[3] A. Thorsted et al. "Model-based assessment of neutrophil-mediated phagocytosis and digestion of bacteria across in vitro and in vivo studies". en. In: *CPT Pharmacometrics Syst Pharmacol* 12.12 (Dec. 2023), pp. 1972–1987. DOI: 10.1002/psp4.13046.

[4] R. W. Watson et al. "Neutrophils undergo apoptosis following ingestion of Escherichia coli." en. In: *J Immunol* 156.10 (May 1996), pp. 3986–3992. DOI: 10.4049/jimmunol.156.10.3986.

[5] J. Murray et al. "Regulation of Neutrophil Apoptosis by Tumor Necrosis Factor-alpha: Requirement for TNFR55 and TNFR75 for Induction of Apoptosis In Vitro". en. In: *Blood* 90.7 (Oct. 1997), pp. 2772–2783. DOI: 10.1182/blood.V90.7.2772.

[6] M. Dubey et al. "Nitric oxide-mediated apoptosis of neutrophils through caspase-8 and caspase-3-dependent mechanism". en. In: *Cell Death Dis* 7.9 (Sept. 2016), e2348–e2348. DOI: 10.1038/cddis.2016.248.

[7] N. Perskvist et al. "*Mycobacterium tuberculosis* Promotes Apoptosis in Human Neutrophils by Activating Caspase-3 and Altering Expression of Bax/Bcl-xL Via an Oxygen-Dependent Pathway". en. In: *J Immunol* 168.12 (June 2002), pp. 6358–6365. DOI: 10.4049/jimmunol.168.12.6358.

[8] G. Engelich, M. White, and K. L. Hartshorn. "Neutrophil survival is markedly reduced by incubation with influenza virus and *Streptococcus pneumoniae* : role of respiratory burst". en. In: *J Leukoc Biol* 69.1 (Jan. 2001), pp. 50–56. DOI: 10.1189/jlb.69.1.50.

[9] D. L. Borjesson et al. "Insights into Pathogen Immune Evasion Mechanisms: *Anaplasma phagocytophilum* Fails to Induce an Apoptosis Differentiation Program in Human Neutrophils". en. In: *J Immunol* 174.10 (May 2005), pp. 6364–6372. DOI: 10.4049/jimmunol.174.10.6364.

[10] K.-S. Choi, J. T. Park, and J. S. Dumler. "*Anaplasma phagocytophilum* Delay of Neutrophil Apoptosis through the p38 Mitogen-Activated Protein Kinase Signal Pathway". en. In: *Infect Immun* 73.12 (Dec. 2005), pp. 8209–8218. DOI: 10.1128/IAI.73.12.8209-8218.2005.

[11] J. Bylund et al. "*Burkholderia cenocepacia* Induces Neutrophil Necrosis in Chronic Granulomatous Disease". en. In: *J Immunol* 174.6 (Mar. 2005), pp. 3562–3569. DOI: 10.4049/jimmunol.174.6.3562.

[12] G. Van Zandbergen et al. "*Chlamydia pneumoniae* Multiply in Neutrophil Granulocytes and Delay Their Spontaneous Apoptosis". en. In: *J Immunol* 172.3 (Feb. 2004), pp. 1768–1776. DOI: 10.4049/jimmunol.172.3.1768.

[13] H. Lundqvist-Gustafsson et al. "Involvement of p38-mitogen-activated protein kinase in staphylococcus aureus-induced neutrophil apoptosis". en. In: *J Leukoc Biol* 70.4 (Oct. 2001), pp. 642–648. DOI: 10.1189/jlb.70.4.642.

[14] S. D. Kobayashi et al. "Bacterial pathogens modulate an apoptosis differentiation program in human neutrophils". en. In: *Proc Natl Acad Sci USA* 100.19 (Sept. 2003), pp. 10948–10953. DOI: 10.1073/pnas.1833375100.

[15] A. Yamamoto et al. "Role of reactive oxygen species in neutrophil apoptosis following ingestion of heat-killed *Staphylococcus aureus*". en. In: *Clin Exp Immunol* 129.3 (Aug. 2002), pp. 479–484. DOI: 10.1046/j.1365-2249.2002.01930.x.

[16] G. Ricevuti et al. "In vivo andin vitro HeNe laser effects on phagocyte functions". en. In: *Inflammation* 13.5 (Oct. 1989), pp. 507–527. DOI: 10.1007/BF00916758.

[17] K. Adolfsson et al. "Direct comparison between in vivo and in vitro microsized particle phagocytosis assays in Drosophila melanogaster". en. In: *Toxicol In Vitro* 46 (Feb. 2018), pp. 213–218. DOI: 10.1016/j.tiv.2017.10.014.

[18] D. Ehlers et al. "Comparison of in vivo and in vitro phagocytosis inGalleria mellonella L." en. In: *Parasitol Res* 78.4 (1992), pp. 354–359. DOI: 10.1007/BF00937096.

[19] J. A. Connelly and K. Walkovich. "Diagnosis and therapeutic decision-making for the neutropenic patient". en. In: *Hematology* 2021.1 (Dec. 2021), pp. 492–503. DOI: 10.1182/hematology.2021000284.

[20] N. S. Potter and C. V. Harding. "Neutrophils Process Exogenous Bacteria Via an Alternate Class I MHC Processing Pathway for Presentation of Peptides to T Lymphocytes". en. In: *J Immunol* 167.5 (Sept. 2001), pp. 2538–2546. DOI: 10.4049/jimmunol.167.5.2538.

[21] H. Hülsheger et al. "Determination of maximal bactericidal activity in human granulocytes". en. In: *Blut* 50.3 (Mar. 1985), pp. 169–178. DOI: 10.1007/BF00320073.

[22] P. C. J. Leijh, M. T. Van Den Barselaar, and R. Van Furth. “Kinetics of Phagocytosis and Intracellular Killing of *Staphytococcus aureus* and *Escherichia coli* by Human Monocytes”. en. In: *Scand J Immunol* 13.2 (Feb. 1981), pp. 159–174. DOI: 10.1111/j.1365-3083.1981.tb00122.x.

[23] M. Nagl et al. “Phagocytosis and Killing of Bacteria by Professional Phagocytes and Dendritic Cells”. en. In: *Clin Vaccine Immunol* 9.6 (Nov. 2002), pp. 1165–1168. DOI: 10.1128/CDLI.9.6.1165-1168.2002.

[24] R. Bjerknes, O. D. Laerum, and S. Ødegaard. “Impaired bacterial degradation by monocytes and macrophages from a patient with treated Whipple’s disease”. en. In: *Gastroenterology* 89.5 (Nov. 1985), pp. 1139–1146. DOI: 10.1016/0016-5085(85)90221-5.

[25] P. K. Peterson et al. “Kinetics of Phagocytosis and Bacterial Killing by Human Polymorphonuclear Leukocytes and Monocytes”. en. In: *J Infect Dis* 136.4 (Oct. 1977), pp. 502–509. DOI: 10.1093/infdis/136.4.502.

[26] G. Zini. “Abnormalities in leukocyte morphology and number”. en. In: *Blood and Bone Marrow Pathology*. Elsevier, 2011, pp. 247–261. DOI: 10.1016/B978-0-7020-3147-2.00016-X.

[27] M. D. Martin et al. “CD115+ monocytes protect microbially experienced mice against E. coli-induced sepsis”. en. In: *Cell Rep* 42.11 (Nov. 2023), p. 113345. DOI: 10.1016/j.celrep.2023.113345.
