# Supplementary material for: A mechanism-based model of immune status effects on antibiotic PK/PD targets in bacteremia
Source: J Pharmacokinet Pharmacodyn. 2026 Sep 22;53(6):53. doi: 10.1007/s10928-026-10063-6 (PMC13597578; doi:10.1007/s10928-026-10063-6)
Supplement: Supplementary file 2 [file 10928_2026_10063_MOESM2_ESM.pdf]

A mechanism-based model of immune status effects on antibiotic PK/PD targets in bacteremia

Marinda van de Kreeke[1†], Anh Duc Pham[1†], Michelle Mehciz[1],
Tamara Jordens[1], Marnix G. Uyterlinde[1], Rob C. van Wijk[1]
Linda B.S. Aulin[1], Catherijne A.J. Knibbe[1,2],
Elke H.J. Krekels[1,3], Laura B. Zwep[1], J.G. Coen van Hasselt[1*]

[1]Division of Systems Pharmacology and Pharmacy, Leiden Academic Centre for Drug Research, Leiden, The Netherlands
[2]Department of Clinical Pharmacy, St. Antonius Hospital, Nieuwegein, The Netherlands
[3]Certara Inc, Radnor, PA, USA

[*]Corresponding author. E-mail:
coen.vanhasselt@lacdr.leidenuniv.nl
[†]These authors contributed equally to this work

Journal of Pharmacokinetics and Pharmacodynamics

Natural apoptosis rate of neutrophils

Data

The data contains 53 observations with the neutrophil starting concentration ranging from 3e+05 to 1e+07 cells/mL. No bacteria are added. The observations are collected within time frame from 0 to 66 hour.

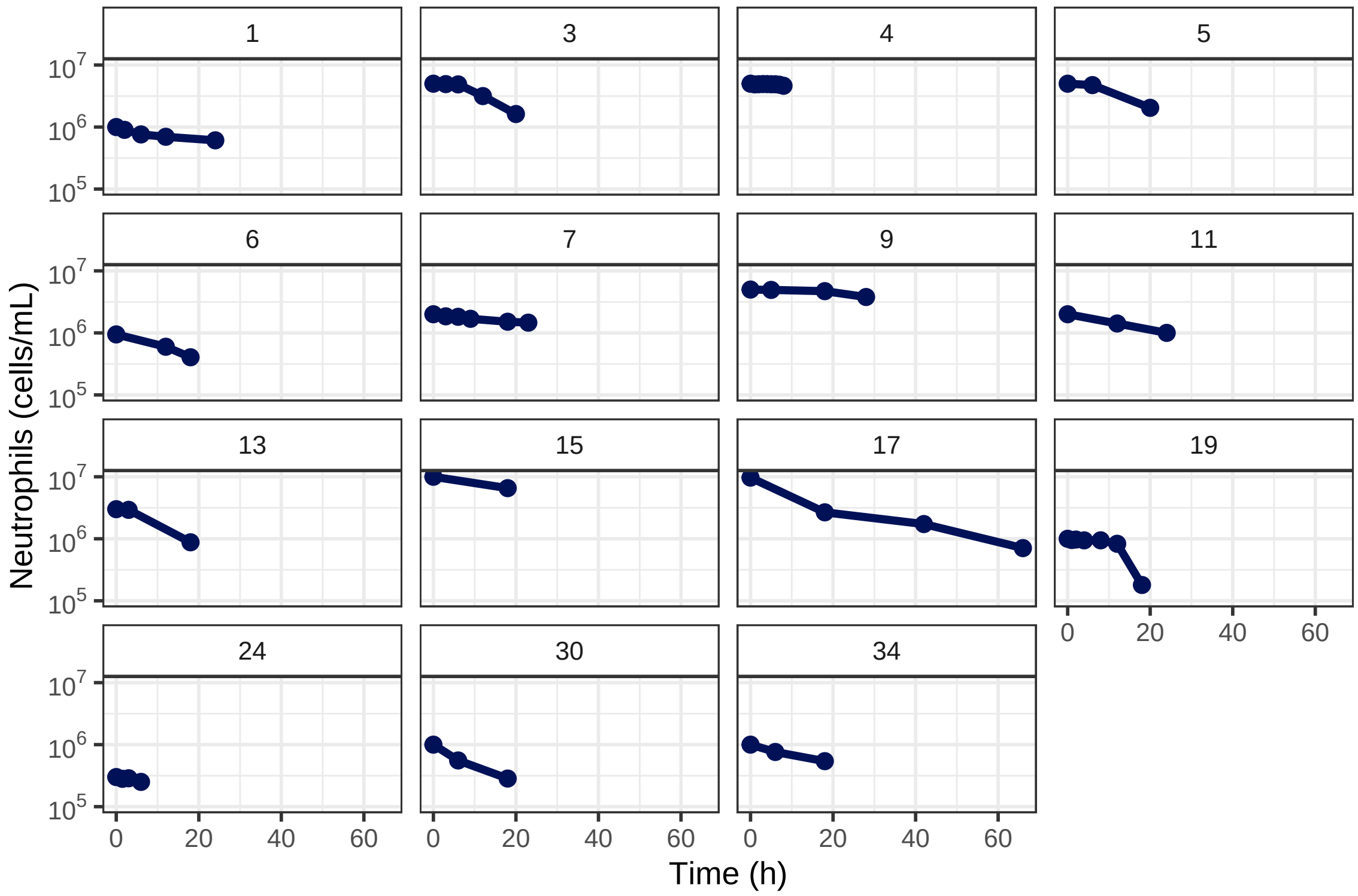

Figure 1: Visualisation of available data for apoptosis of neutrophils

Models

A linear rate constant was assumed for the apoptosis of neutrophils. This rate was estimated as a typical value: the average of the whole population (all studies combined) and then individually, to determine the spread between different strains and experiments. Model evaluation was based on precision and plausibility of parameter estimates and visual predictive checks (VPCs).

Parameter estimation with submodel

Typical parameter value: estimation based on all data The typical parameter estimate of knapop is 0.0371 (9.81 %).

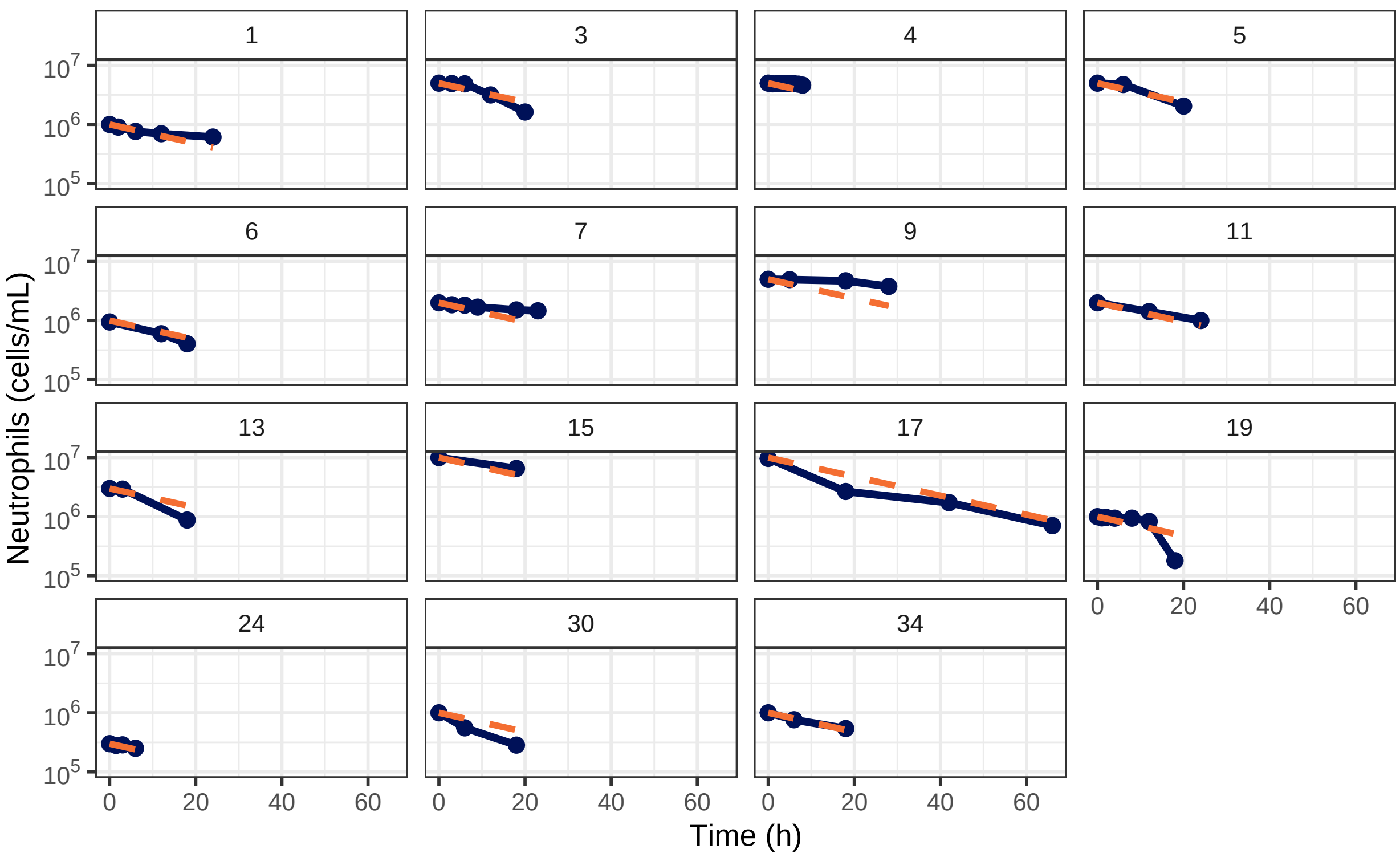

Figure 2: Model fit for the typical apoptosis rate of neutrophils

Range of parameter value: estimation per ID in the data

Table 1: Individual and typical parameter estimates of the apoptosis rate of neutrophils

SID	ID	Estimate	RSE
100	1	0.0238	19.35
101	3	0.0480	21.57
101	4	0.0062	23.05
101	5	0.0418	23.84
102	6	0.0479	7.27
103	7	0.0148	7.82
104	9	0.0078	32.20
105	11	0.0288	NA

SID	ID	Estimate	RSE
106	13	0.0666	14.67
107	15	0.0235	NA
108	17	0.0423	12.04
109	19	0.0612	38.18
111	24	0.0276	17.22
112	30	0.0726	11.48
112	34	0.0352	9.61
NA	Typical	0.0371	9.81

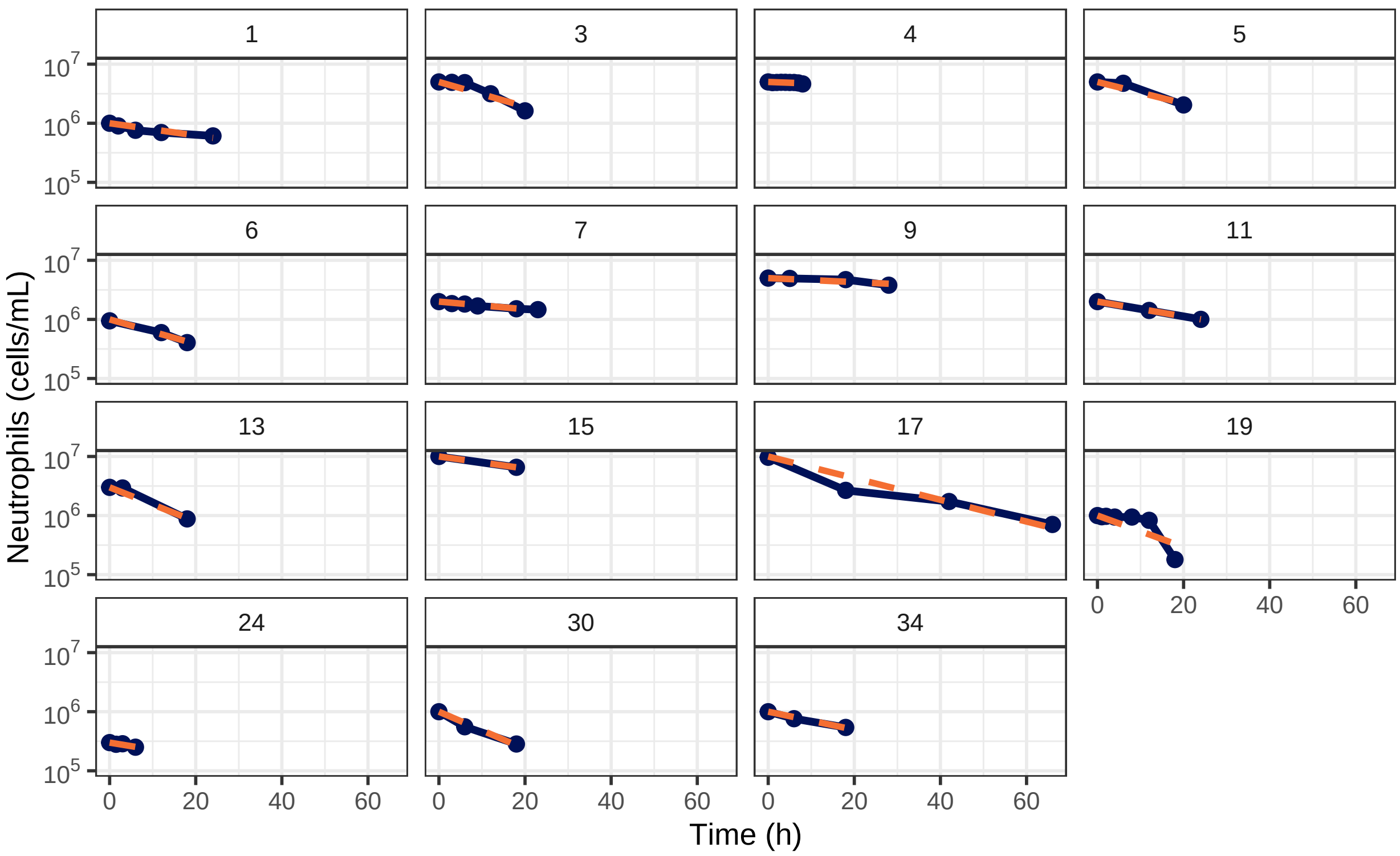

Figure 3: Individual model fit of the apoptosis rate of neutrophils

Conclusion fit of knapop

The typical parameter estimate of the apoptosis rate of neutrophils is 0.0371 (9.81 %). The range of the parameter estimates of knapop is 0.0062 - 0.0726. The fit is acceptable to continue with estimating the phagocytosis-induced cell death rate of neutrophils (knpicd).

knpicd while knapop is fixed

Data

The data contains 113 observations with the neutrophil starting concentration ranging from 3e+05 to 1e+07 cells/mL. The observations are collected within time frame from 0 to 66 hour.

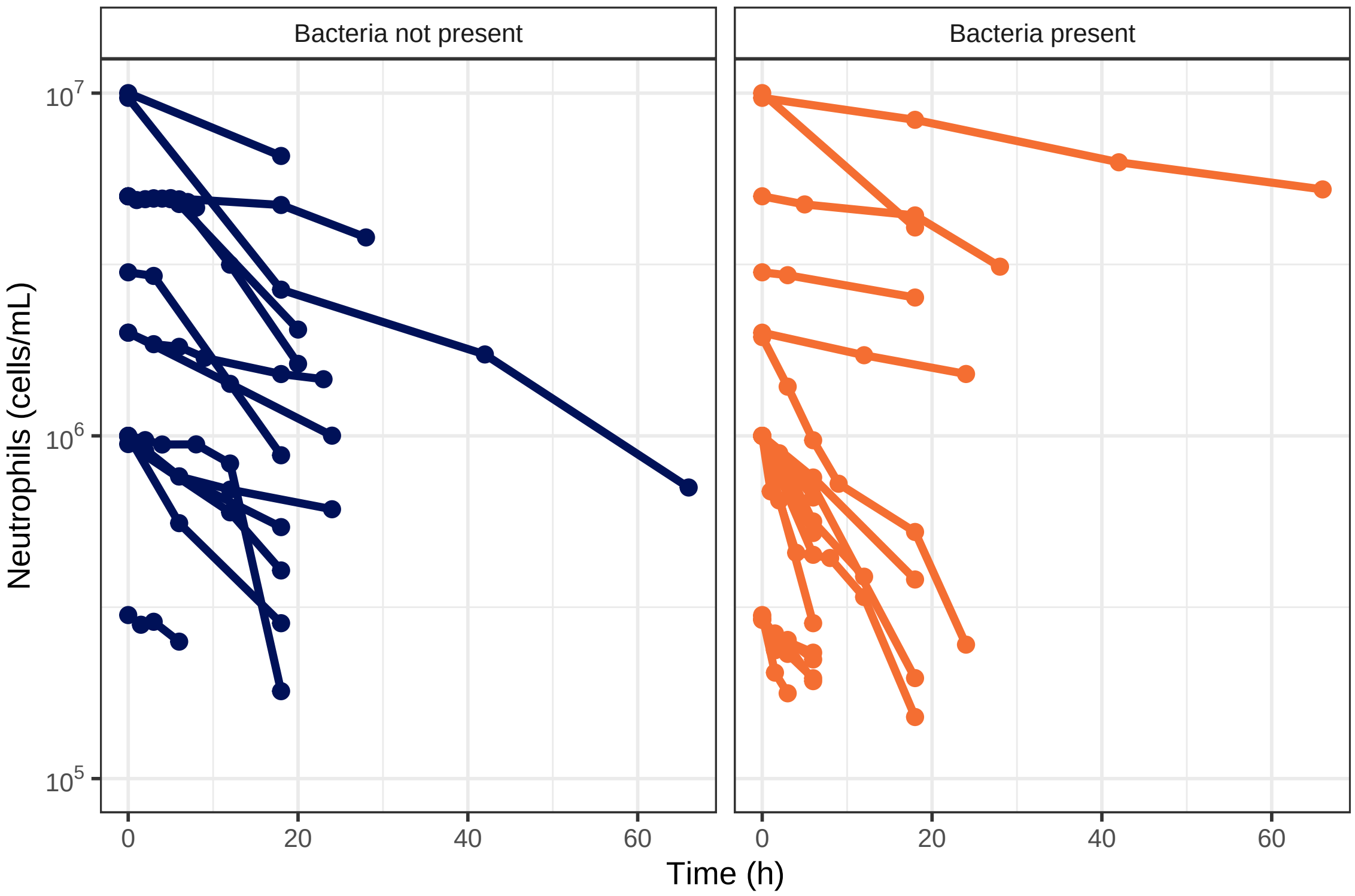

Figure 4: Visualisation of available data for apoptosis and phagocytosis-induced cell death of neutrophils

Models

A linear rate constant was assumed for the apoptosis of neutrophils and additionally also a linear rate constant for the phagocytosis-induced cell death (picd) of neutrophils, which is only added if bacteria are present (indicated by FLAG in the data). The picd rate was estimated as a typical value: the average of the whole population (all studies combined) and then individually, to determine the spread between different strains and experiments. knapop was fixed during the estimation of knpicd. Model evaluation was based on precision and plausibility of parameter estimates and visual predictive checks (VPCs).

Parameter estimation with submodel

Typical parameter value: estimation based on all data The typical parameter estimate of knpicd is 0.00924 (120 %).

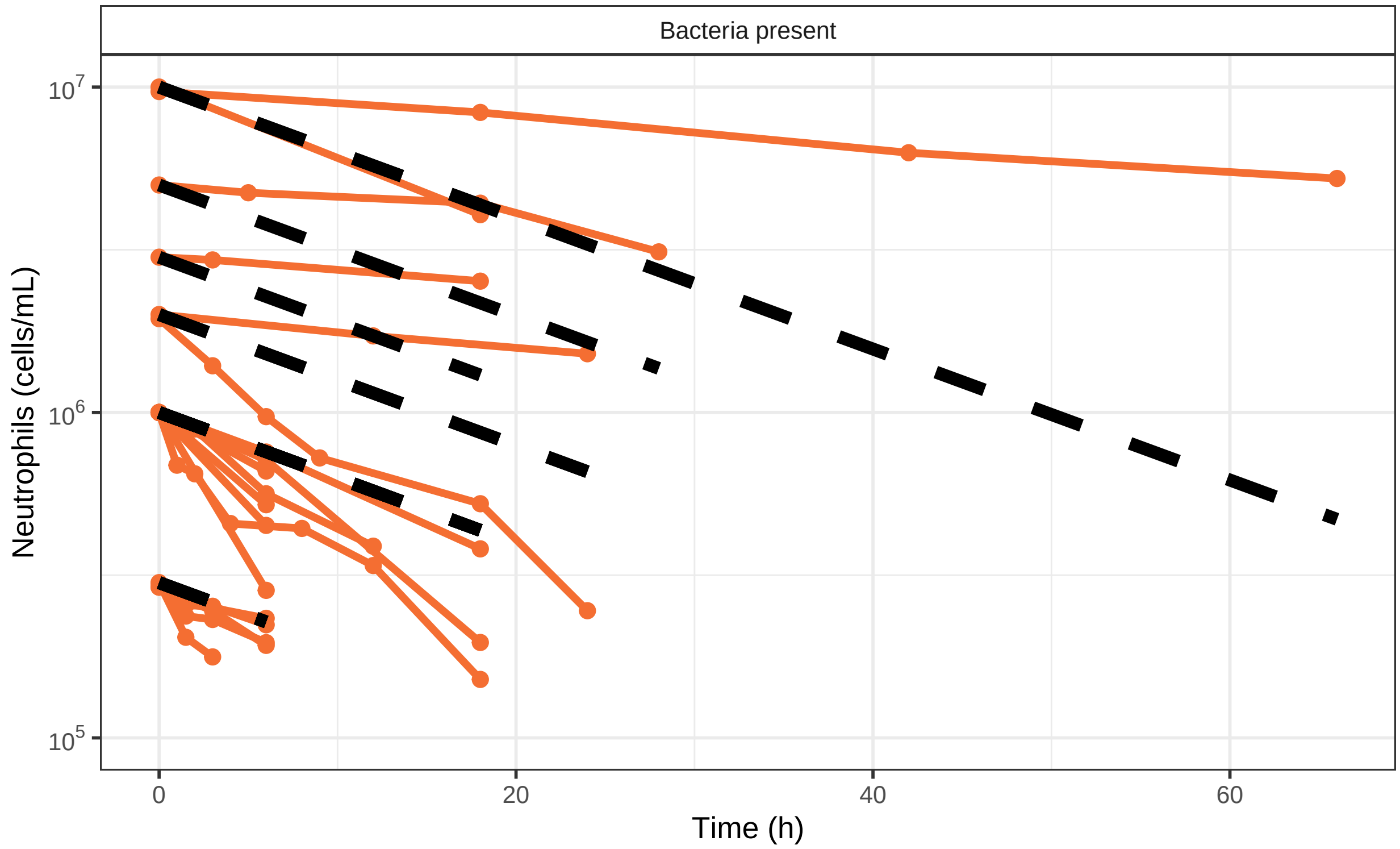

Figure 5: Model fit for the typical picd rate of neutrophils

Range of parameter value: estimation per ID in the data

Table 4: Individual and typical parameter estimates of the phagocytosis-induced cell death (PICD) rate of neutrophils

SID	ID	Estimate	RSE
100	2	0.0447	13.85
103	8	0.0496	14.39
104	10	-0.0231	15.54
105	12	-0.0253	1.37
106	14	-0.0278	1.75
107	16	0.0130	NA
108	18	-0.0269	1.80
109	20	0.0677	19.45
111	25	0.0111	85.19
111	26	0.0165	55.61

SID	ID	Estimate	RSE
111	27	0.0409	33.30
111	28	0.0357	11.93
111	29	0.2963	NA
112	31	0.1854	2.29
112	32	0.1778	15.32
112	33	0.1151	12.60
112	35	0.0940	21.98
112	36	0.0499	20.65
112	37	0.0158	13.18
NA	Typical	0.0092	120.37

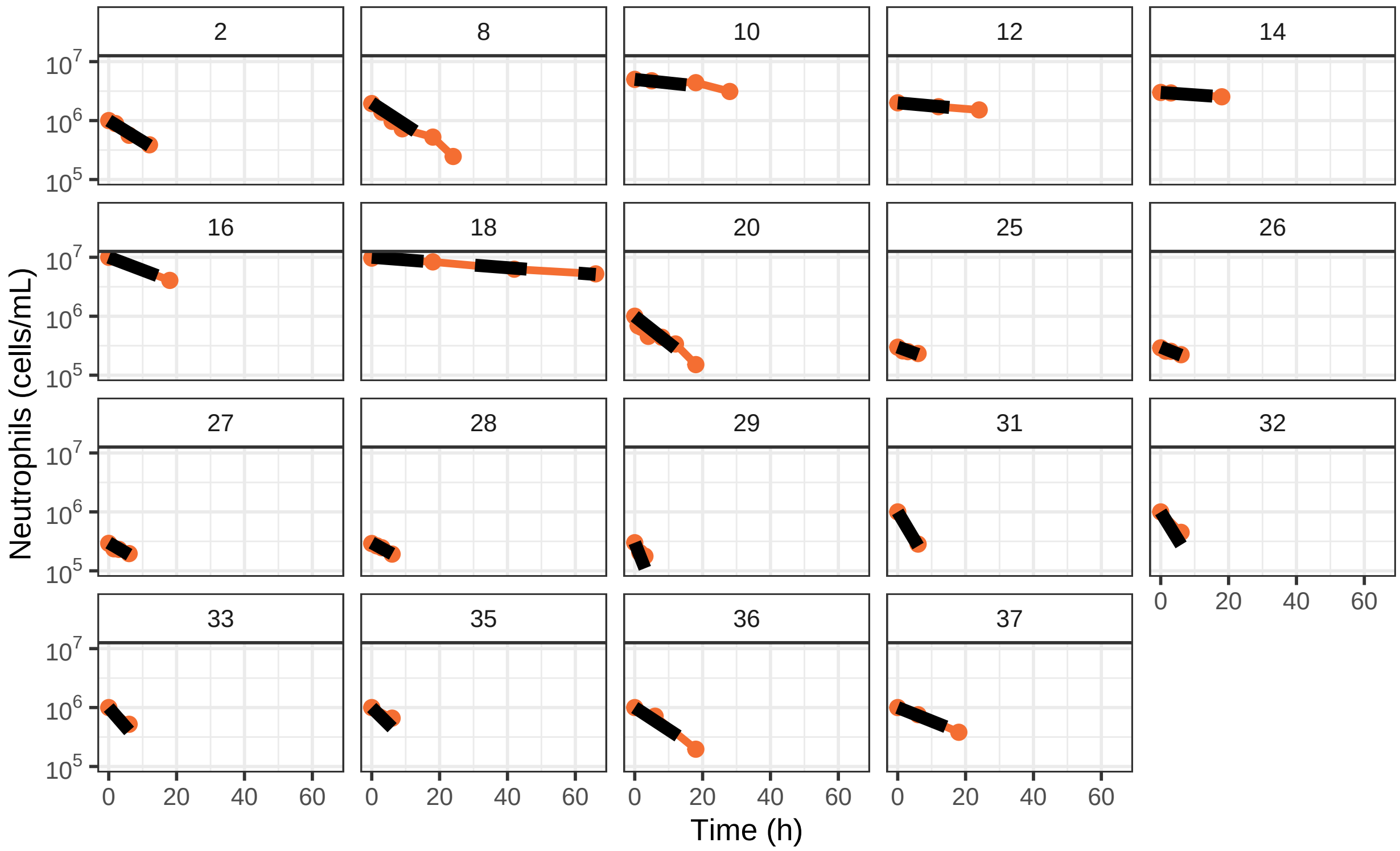

Figure 6: Individual model fit of the phagocytosis-induced cell death rate of neutrophils

Conclusion fit of knpicd

The typical parameter estimate of the phagocytosis-induced cell death rate of neutrophils is 0.00924 (120 %). The range of the parameter estimates of knpicd is -0.0278 - 0.2963. The range is broad because some bacterial strains stimulate bacterial death, while others slow down this process. The typical values will be used for the simulations with the model.
