# Supplementary material for: A mechanism-based model of immune status effects on antibiotic PK/PD targets in bacteremia
Source: J Pharmacokinet Pharmacodyn. 2026 Sep 22;53(6):53. doi: 10.1007/s10928-026-10063-6 (PMC13597578; doi:10.1007/s10928-026-10063-6)
Supplement: Supplementary file 3 [file 10928_2026_10063_MOESM3_ESM.pdf]

A mechanism-based model of immune status effects on antibiotic PK/PD targets in bacteremia

Marinda van de Kreeke[1†], Anh Duc Pham[1†], Michelle Mehciz[1],
Tamara Jordens[1], Marnix G. Uyterlinde[1], Rob C. van Wijk[1]
Linda B.S. Aulin[1], Catherijne A.J. Knibbe[1,2],
Elke H.J. Krekels[1,3], Laura B. Zwep[1], J.G. Coen van Hasselt[1*]

[1]Division of Systems Pharmacology and Pharmacy, Leiden Academic Centre for Drug Research, Leiden, The Netherlands
[2]Department of Clinical Pharmacy, St. Antonius Hospital, Nieuwegein, The Netherlands
[3]Certara Inc, Radnor, PA, USA

[*]Corresponding author. E-mail:
coen.vanhasselt@lacdr.leidenuniv.nl
[†]These authors contributed equally to this work

Journal of Pharmacokinetics and Pharmacodynamics

Digestion rate of monocytes

Data

The data contains 68 observations with the pathogen concentration range of 9.1e+04 - 1.5e+08 CFU/mL vs only one cell concentration of 5e+06 cells/mL. The observations are collected within time frame from 0 to 2 hour.

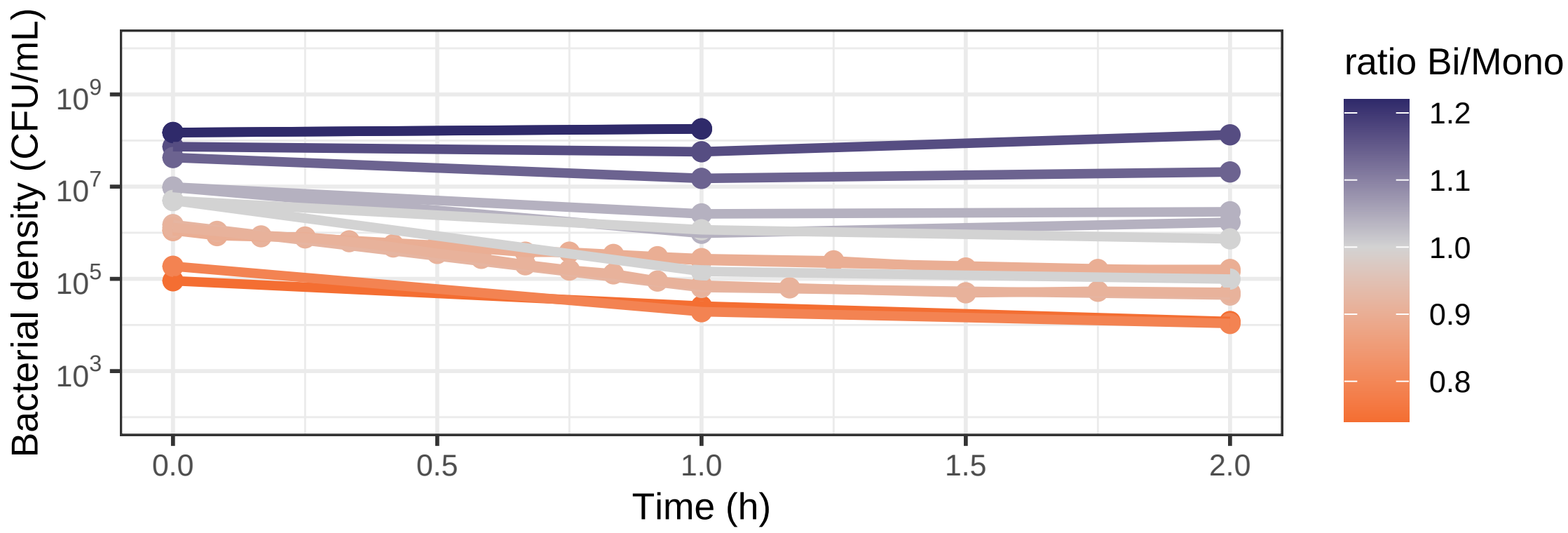

Figure 1: Visualisation of available data for digestion by monocytes

Models

The data from the first and second hours are modeled sequentially. Initially, the elimination/digestion rate is estimated, focusing on the first-order digestion rate of pathogens which are intracellular. The relationship between the number of intracellular pathogens and the digestion rate is investigated using linear, linear with power and Emax functions. We assume that similar to the observed dependancy of neutrophil digestion on the concentration of neutrophils, a similar dependency is seen here with the monocyte digestion on the concentration of monocytes. Because these experiments only included one concentration of monocytes, we assume the same value for M50_dig as we have for N50_dig. The plateau observed in the data after the first hour of the experiment is then described by the degradation rate relative to the digestion rate over time. Residual unexplained variability was assessed using a proportional relationship. Model evaluation and discrimination were based on the objective function value (ΔOFV > 3.84 for nested models with 1 df, = 0.05), goodness-of-fit plots, precision and plausibility of parameter estimates, and visual predictive checks (VPCs).

Modelling results 0-1 hour

Table 1: Model fit and parameters for digestion rate of monocytes on 0-1 hour data

Elements	Slope	Slope_ratio	Slope_ratio_power	Emax_ratio
OFV	-75.32	-95.2	-75.33	-117.65
DOFV	0	-19.89	-0.01	-42.34
Digestion rate	3.15(11.5)	9.79(20.8)	3.16(11.7)	3.8(7.01)
Slope	NA	4.02(30.2)	4(1.63e+03)	NA
Power	NA	NA	4.99(242)	NA
R50	NA	NA	NA	1.11(1.87)
Hill	NA	NA	NA	42.6(90.9)
RUV	3.98e+06	0.233	0.286	0.186
IIV	NA	NA	NA	NA

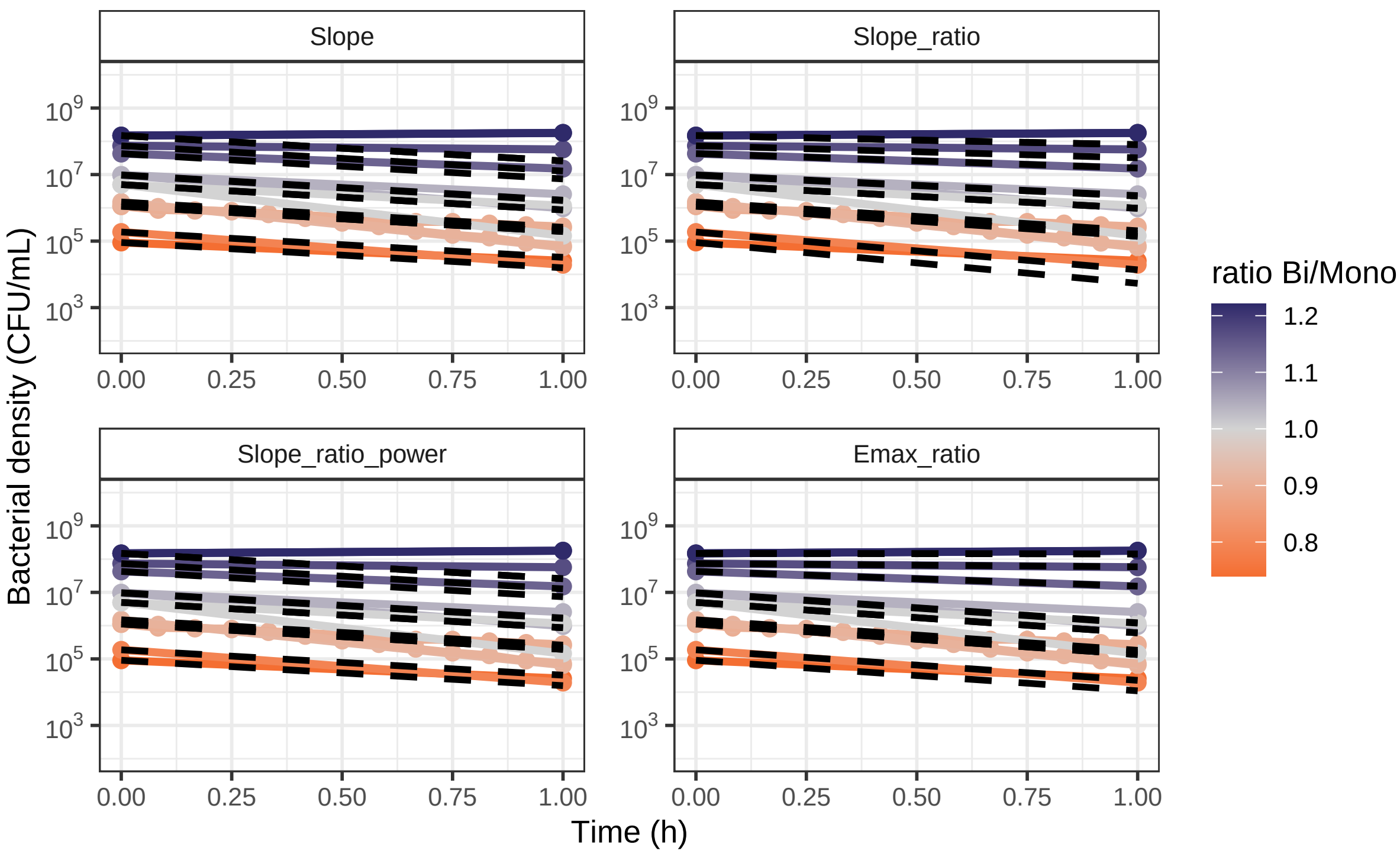

Figure 2: Model fit for digestion rate of monocytes on 0-1 hour data

Conclusion model fit 0-1 hour

The base model with digestion rate independent from the ratio showed the worst fit. Addition of ratio dependent showed significant model fit than the base model (ΔOFV = -19.8883077). The power function improved the model but the improvement is not significant (ΔOFV = 19.8737513). The Emax relationship based on the ratio is the best model (ΔOFV = -42.338611)

Modelling results 0-2 hours

Table 2: Model fit and parameters for digestion rate of monocytes on 0-2 hour data

Elements	Emax	Emax_decay
OFV	-97.59	-121.41
DOFV	0	-23.81
Digestion rate	2.97(9.5)	6.23(20.5)
R50	1.05(3.64)	1.05(2.97)
Hill	14.1(33.3)	14(31.8)
Decay	NA	1.1(33.6)
RUV	0.297	0.249
IIV	NA	NA

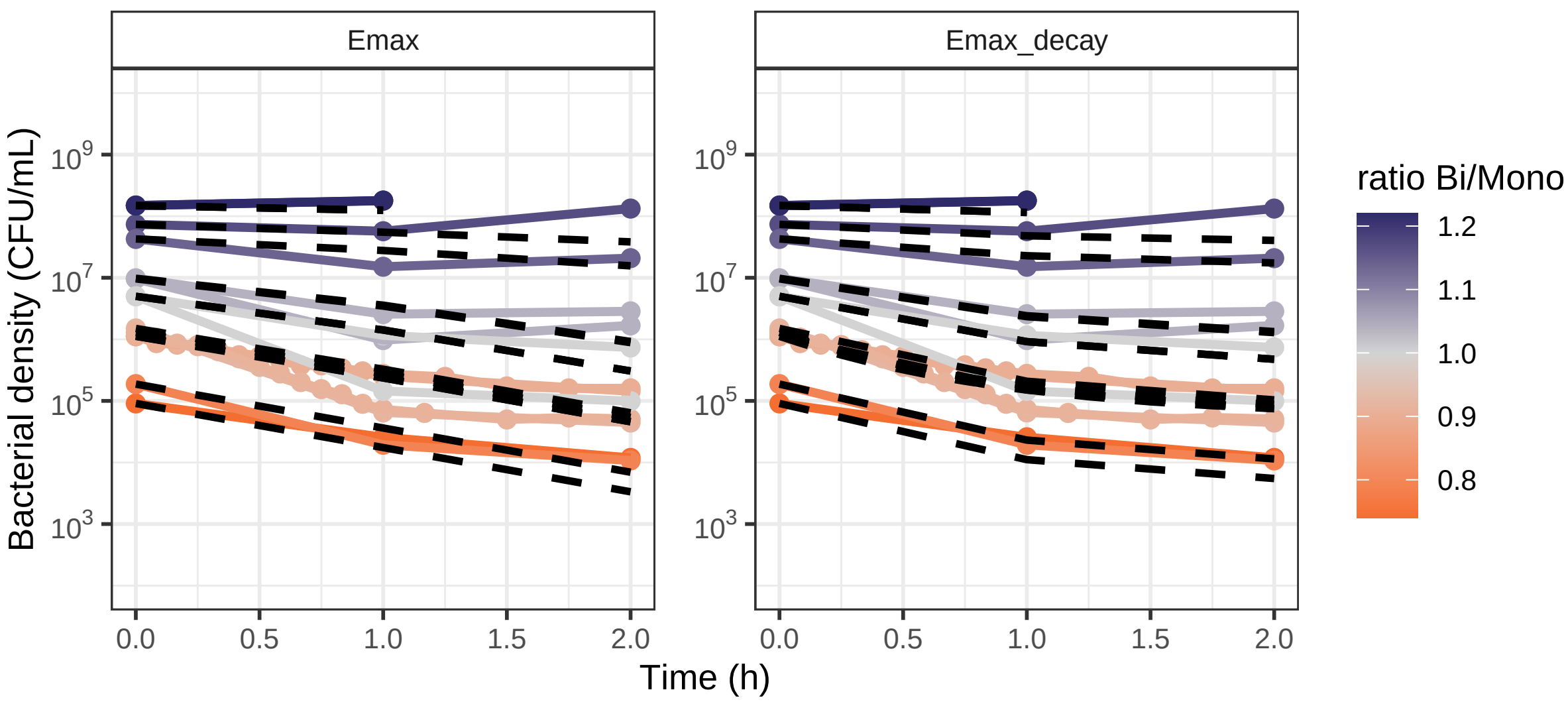

Figure 3: Model fit for digestion rate of monocytes on 0-2 hour data

Conclusion model fit 0-2 hours

The Emax function based on the ratio between bacteria and monocytes with the addition of a decay over time describes the digestion rate of the data best.

Phagocytosis rate of monocytes

Data

The data contains 121 observations with the pathogen concentration range of 5.1e+03 - 4.4e+09 CFU/mL and monocyte cell concentration range of 5e+04 - 5e+06 cells/mL. The observations are collected within time frame from 0 to 2 hour.

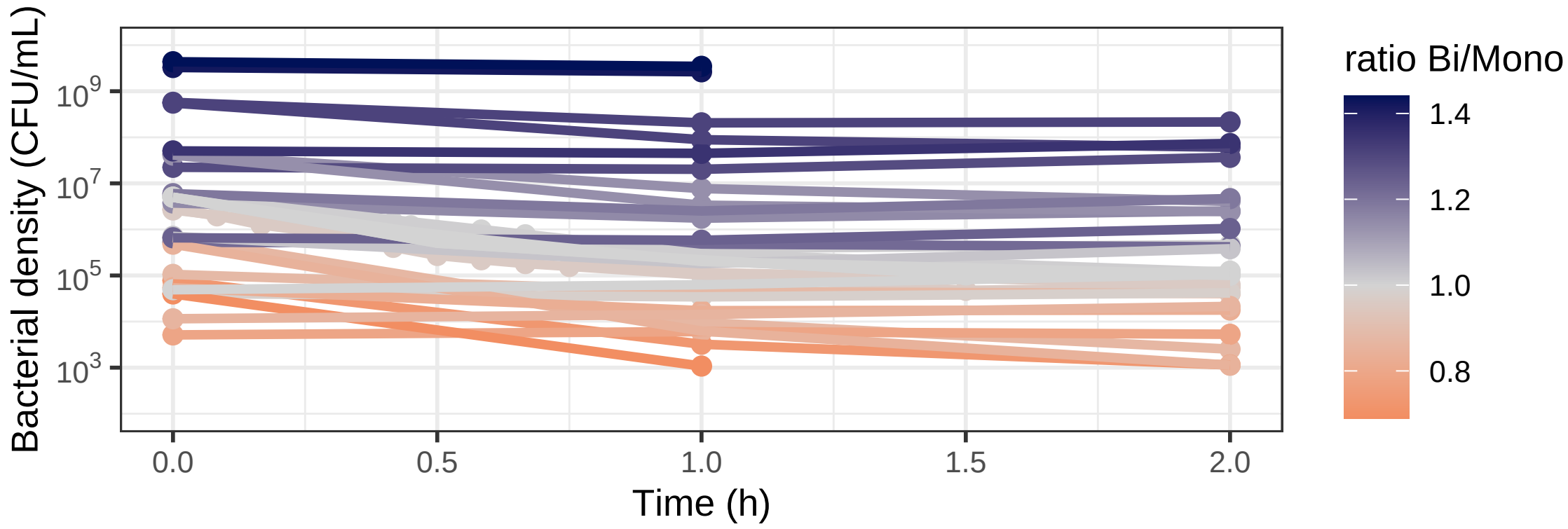

Figure 4: Visualisation of available data for phagocytosis by monocytes

Models

The data from the first and second hours are modeled sequentially. Initially, the phagocytosis rate is estimated, focusing on the first-order phagocytosis rate of pathogens which are extracellular. The relationship between the number of intracellular pathogens and the phagocytosis rate is investigated using linear, linear with power and Emax functions. The assumption is made that bacteria are growing with a constant net growth rate of 1.451 /h (in vitro average of multiple species). A combined model is used of phagocytosis and digestion, where the digestion parameters are fixed on the estimates from previous research. We assume that similar to the observed dependancy of neutrophil phagocytosis on the concentration of neutrophils, a similar dependency is seen here with the monocyte phagocytosis on the concentration of monocytes.

The plateau observed in the data after the first hour of the experiment is then described by the degradation rate relative to the phagocytosis rate over time. Residual unexplained variability was assessed using a proportional relationship. Model evaluation and discrimination were based on the objective function value (ΔOFV $>$ 3.84 for nested models with 1 df, = 0.05), goodness-of-fit plots, precision and plausibility of parameter estimates, and visual predictive checks (VPCs).

Modelling results 0-1 hour

Table 3: Model fit and parameters for phagocytosis rate of monocytes on 0-1 hour data

Elements	Slope	Slope_ratio	Slope_ratio_power	Emax_ratio
OFV	-109.67	-112.89	-194.42	-208.11
DOFV	0	-3.22	-84.74	-98.44
Phagocytosis rate	4.97(4.84)	6.68(13.1)	5.6(3.39)	5.98(5.55)
M50_phag	6.92e+05(FIXED)	6.92e+05(FIXED)	6.92e+05(FIXED)	6.92e+05(FIXED)
Slope	NA	1.42(48.6)	0.0928(105)	NA
Power	NA	NA	12(30)	NA
R50	NA	NA	NA	1.24(2.99)
Hill	NA	NA	NA	9.66(27.7)
RUV	3.98e+06	1.1	3.98e+06	3.98e+06
IIV	NA	NA	NA	NA

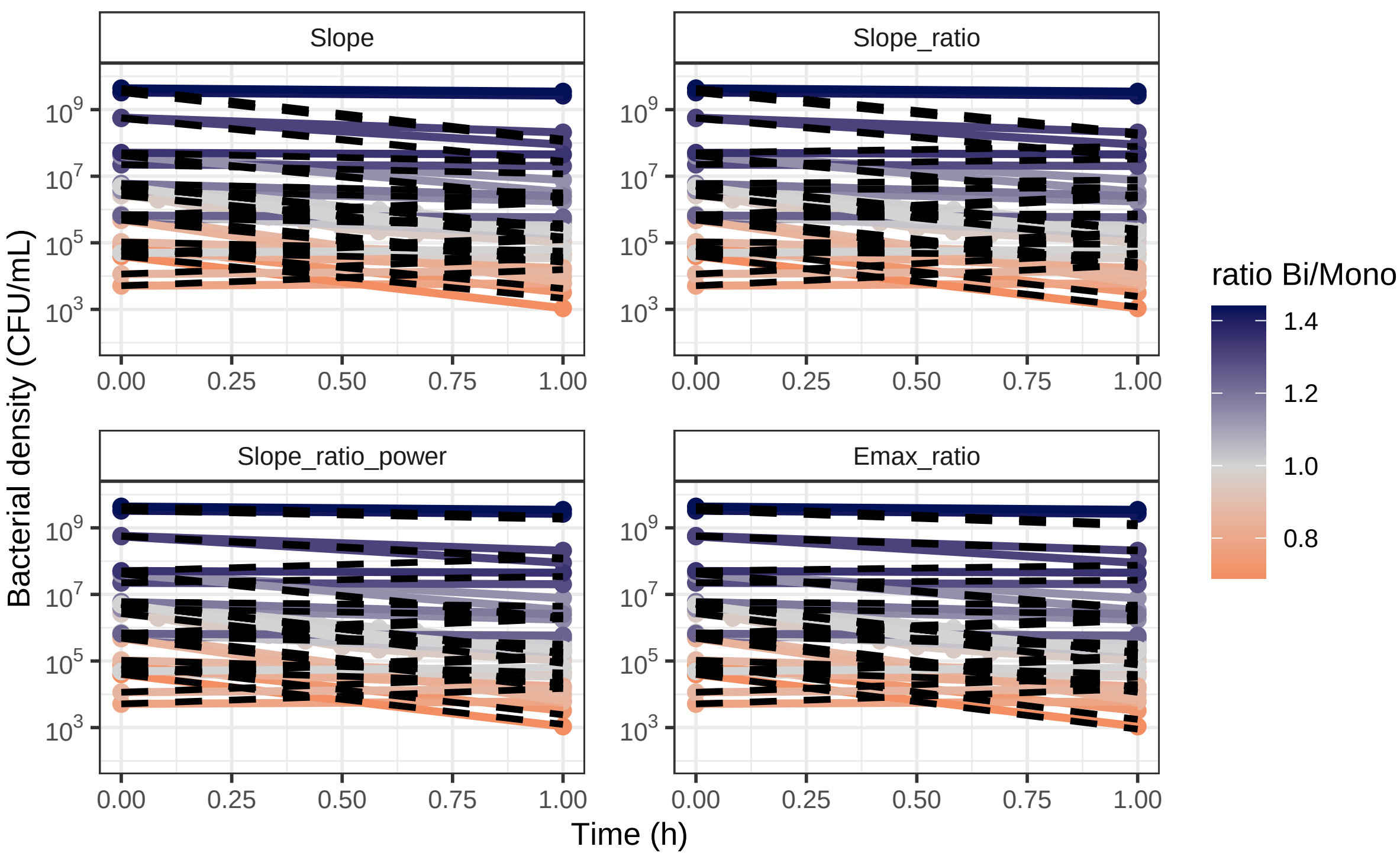

Figure 5: Model fit for phagocytosis rate of monocytes on 0-1 hour data

Conclusion model fit 0-1 hour

The base model with phagocytosis rate independent from the ratio showed the worst fit. Addition of ratio dependent showed significant model fit than the base model (ΔOFV = -3.2158336). The power function improved the model but the improvement is not significant (ΔOFV = -81.5289621). The Emax relationship based on the ratio is the best model (ΔOFV = -98.4355461)

Modelling results 0-2 hours

Table 4: Model fit and parameters for phagocytosis rate of monocytes on 0-2 hour data

Elements	Emax	Emax_decay	Emax_decay_est
OFV	-139.89	-175.47	-220.36
DOFV	0	-35.58	-80.47
Phagocytosis rate	5.19(12)	6.19(8.89)	7.78(10.9)
M50_phag	6.92e+05(FIXED)	6.92e+05(FIXED)	2.73e+05(4.12)
R50	1.28(5.69)	1.32(3.68)	1.25(3.77)
Hill	5.79(40.8)	8.04(28.1)	5.54(26.7)
Decay	NA	0.35(31.7)	0.657(17.5)
RUV	NA	NA	NA
IIV	NA	NA	NA

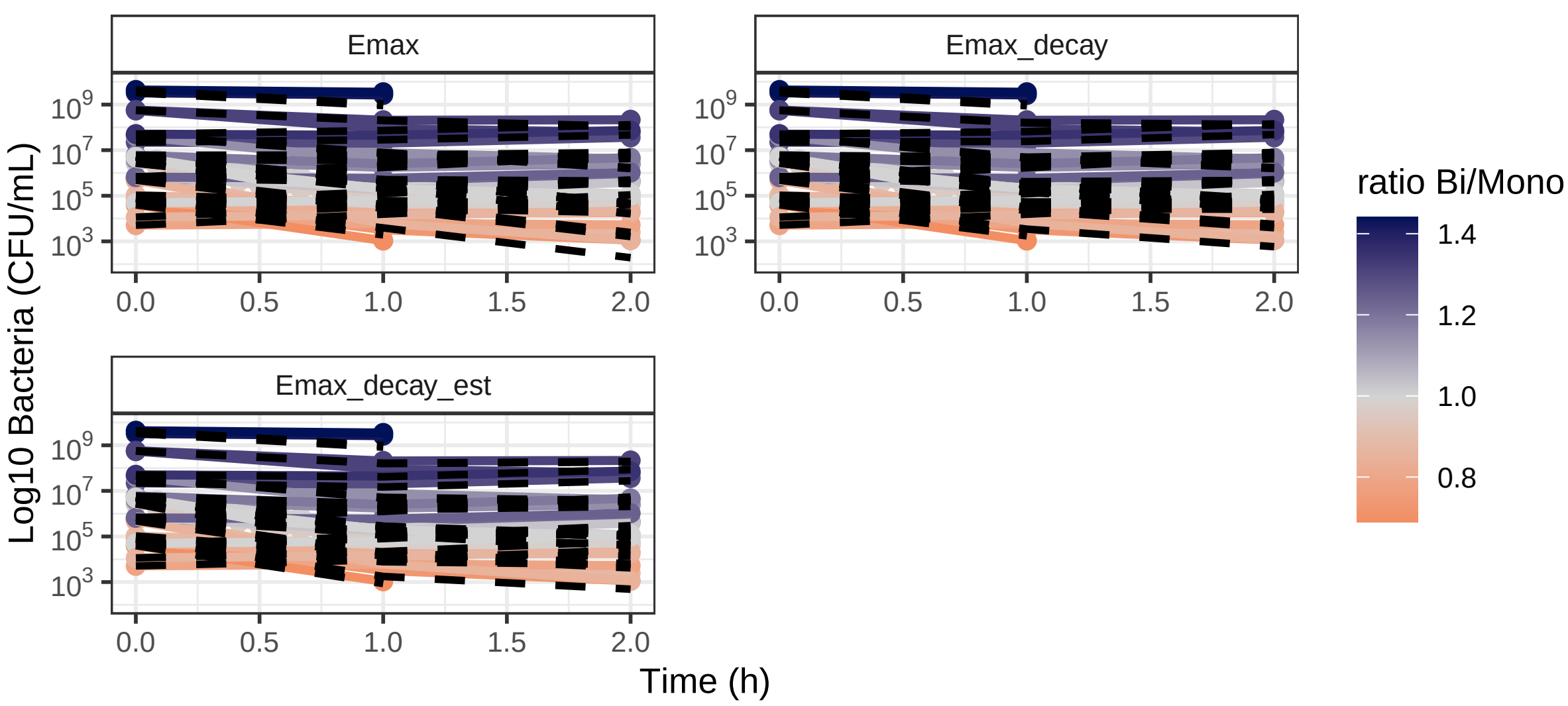

Figure 6: Model fit for phagocytosis rate of monocytes on 0-2 hour data

Conclusion model fit 0-2 hours

The Emax function based on the ratio between bacteria and monocytes with the addition of a decay over time describes the phagocytosis rate best.

Adapt estimations of phagocytosis and digestion rate of monocytes

Data

The data contains 217 observations with the pathogen concentration range of 5.1e+03 - 4.4e+09 CFU/mL and monocyte cell concentration range of 5e+04 - 5e+06 cells/mL. The observations are collected within time frame from 0 to 4 hour.

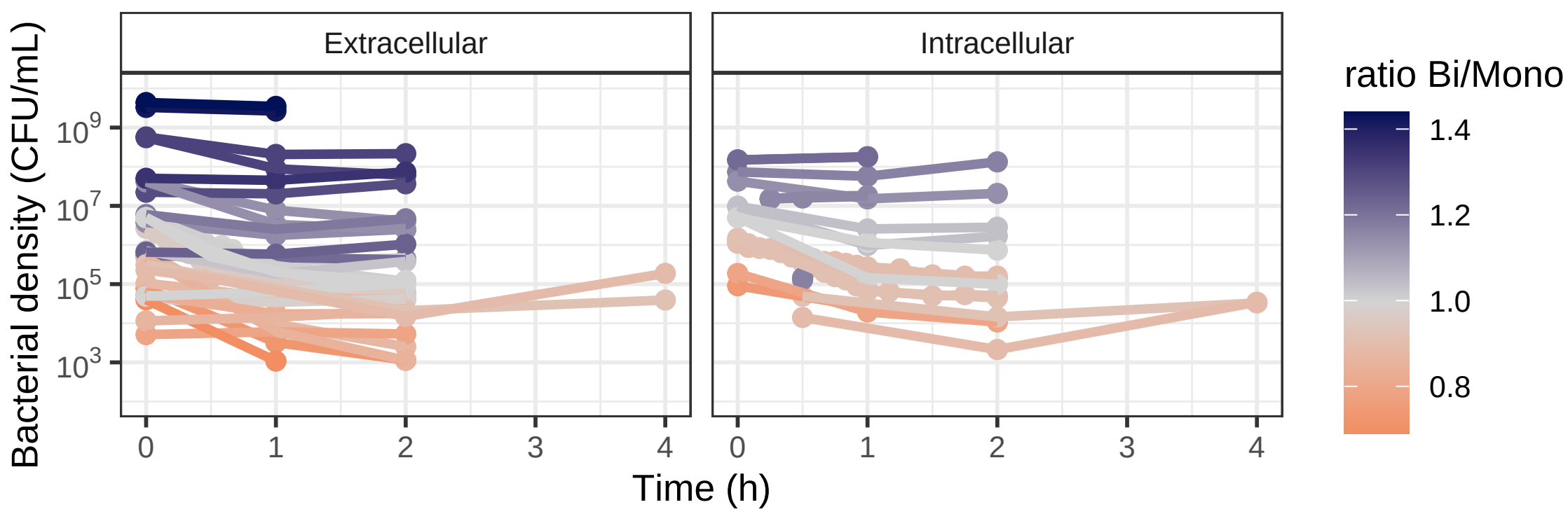

Figure 7: Visualisation of available data for digestion and phagocytosis by monocytes

Models

Previously, the phagocytosis and digestion rate of monocytes were estimated separately. The phagocytosis rate was estimated to be 7.78 /h with a Hill coefficient of 5.54, B_M50 of 1.25 CFU/cell, M50 of 2.73e05 cell/mL and decay rate of 0.657 /h. The digestion rate was estimated to be 6.23 /h with a Hill coefficient of 14, B_M50 of 1.05 CFU/cell, M50 of 3.98e06 cell/mL and decay rate of 1.1 /h.

In this analysis, the phagocytosis and digestion rate of monocytes are re-estimated using the combined data of separate phagocytosis and digestion experiments together with the data of a combined phagocytosis & digestion experiment. The model is fitted with fixed parameters and re-estimated parameters. The model fit is evaluated

Residual unexplained variability was assessed using a proportional relationship. Model evaluation and discrimination were based on the objective function value (ΔOFV $>$ 3.84 for nested models with 1 df, $= 0.05$), goodness-of-fit plots, precision and plausibility of parameter estimates, and visual predictive checks (VPCs).

Modeling results on all in vitro data (-16h timepoint)

Table 5: Model fit and parameters for phagocytosis and digestion rate of monocytes on 0-4 hour data

Elements	fixed_parameters	Re_estimated
OFV	-271.16	-273.27
DOFV	NA	-2.11
Phag_emax	7.78()	7.78(9.4)
M50_phag	2.73e+05()	2.73e+05(23.9)
Phag_R50	1.25()	1.27(3.89)
Phag_Hill	5.54()	5.54(24.1)
Phag_Decay	0.657()	0.692(13.6)
Dig_emax	6.23()	6.23(48.2)
M50_dig	3.98e+06()	3.98e+06(0.0183)
Dig_R50	1.05()	1.06(5.52)
Dig_Hill	14()	14(60)
Dig_Decay	1.1()	0.975(84.9)
RUV_E	0.251	0.251
RUV_I	0.471	0.471

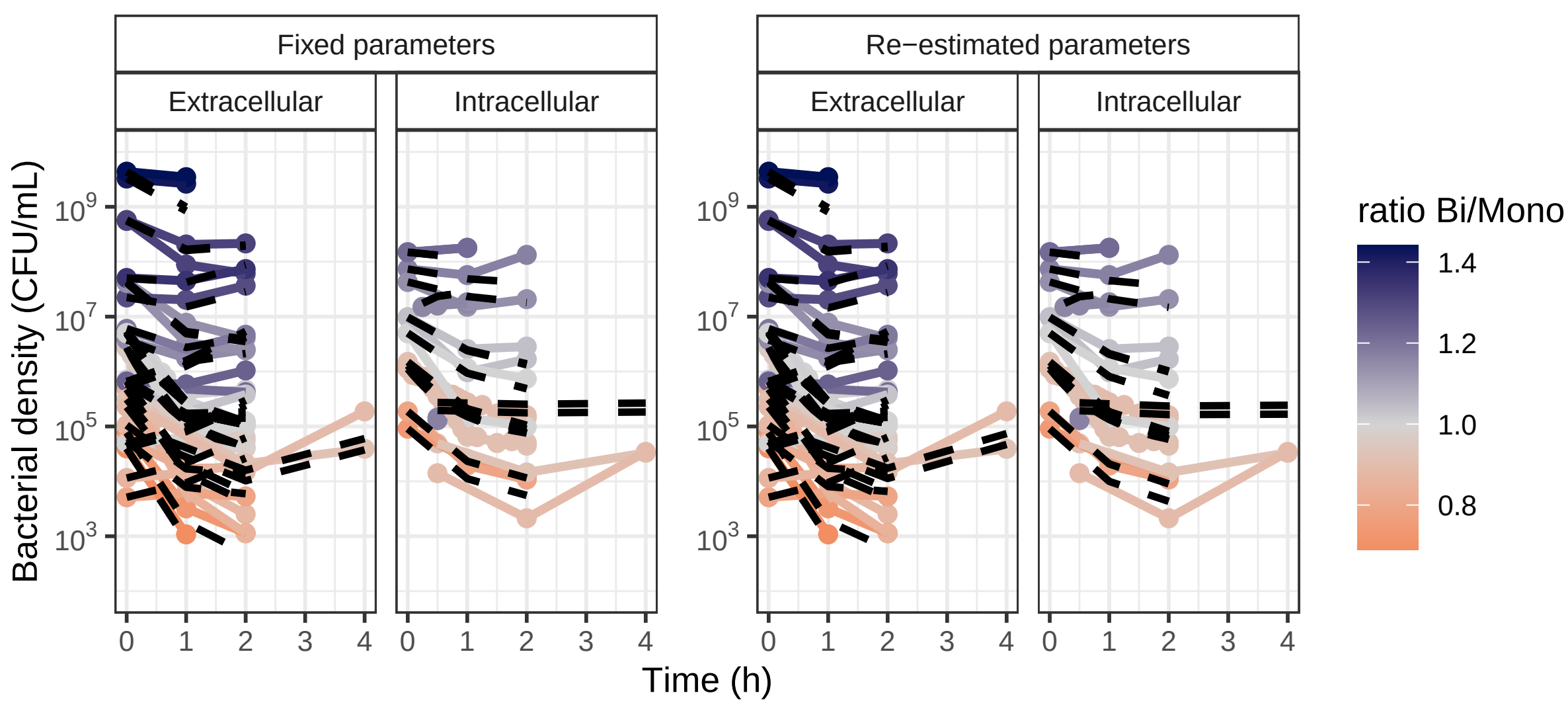

Figure 8: Model fit for phagocytosis and digestion rate of monocytes on 0-4 hour data

Conclusion model fit 0-4 hours

There is not much difference between the model fit with fixed and re-estimated parameters. The visual predictive check also doesn't show much improvement. Therefore, we will keep the separately estimated parameters for phagocytosis and digestion.
