# Supplementary material for: A mechanism-based model of immune status effects on antibiotic PK/PD targets in bacteremia
Source: J Pharmacokinet Pharmacodyn. 2026 Sep 22;53(6):53. doi: 10.1007/s10928-026-10063-6 (PMC13597578; doi:10.1007/s10928-026-10063-6)
Supplement: Supplementary file 4 [file 10928_2026_10063_MOESM4_ESM.pdf]

A mechanism-based model of immune status effects on antibiotic PK/PD targets in bacteremia

Marinda van de Kreeke[1†], Anh Duc Pham[1†], Michelle Mehciz[1],
Tamara Jordens[1], Marnix G. Uyterlinde[1], Rob C. van Wijk[1]
Linda B.S. Aulin[1], Catherijne A.J. Knibbe[1,2],
Elke H.J. Krekels[1,3], Laura B. Zwep[1], J.G. Coen van Hasselt[1*]

[1]Division of Systems Pharmacology and Pharmacy, Leiden Academic Centre for Drug Research, Leiden, The Netherlands
[2]Department of Clinical Pharmacy, St. Antonius Hospital, Nieuwegein, The Netherlands
[3]Certara Inc, Radnor, PA, USA

[*]Corresponding author. E-mail:
coen.vanhasselt@lacdr.leidenuniv.nl
[†]These authors contributed equally to this work

Journal of Pharmacokinetics and Pharmacodynamics

In vivo study data

The data contains the following information: CFU measurements at 24hours for four mice, and neutrophil and monocyte concentrations in a separate group of 6 uninfected mice. The data is structured in a way that the model can be fitted to the CFU data, while the neutrophil and monocyte concentrations are used as covariates.

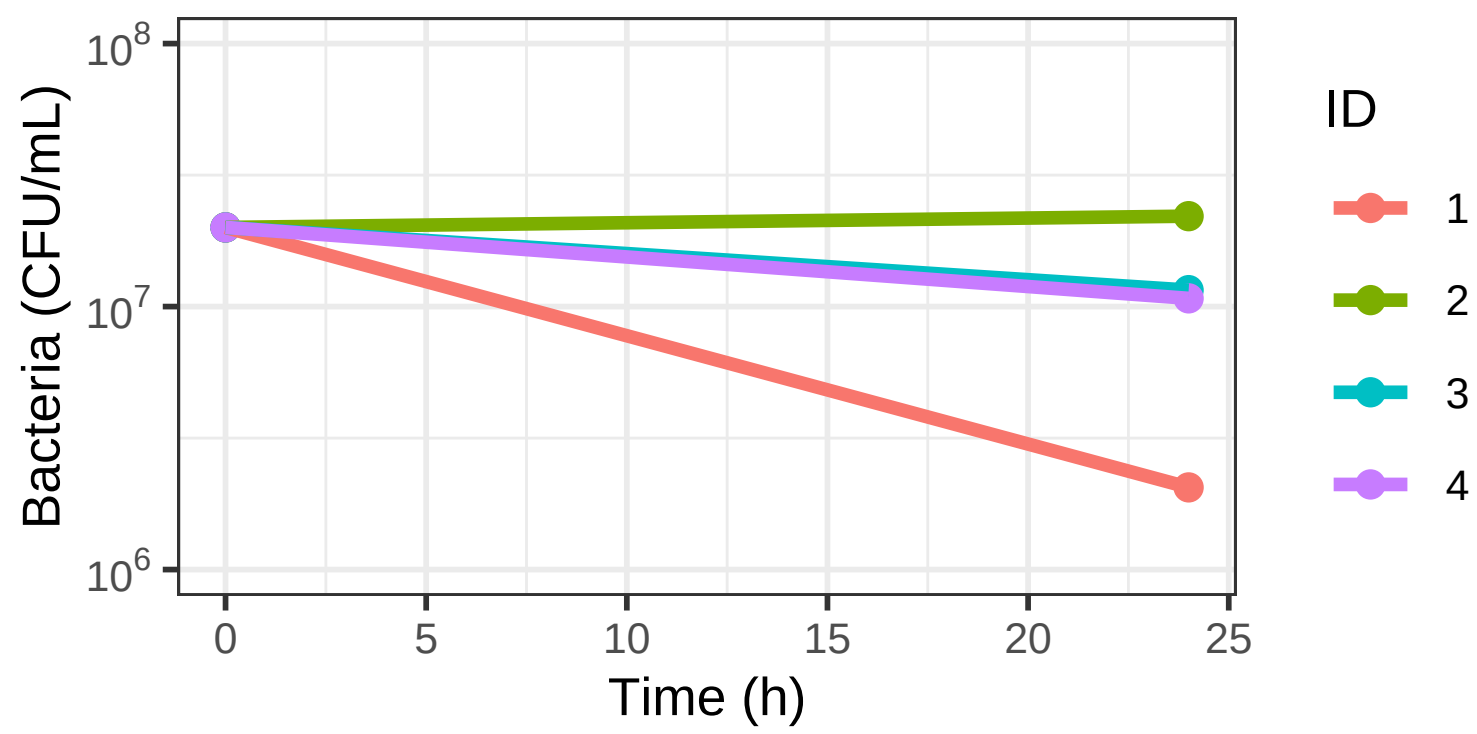

Figure 1: Visualisation of available *in vivo* data. The four mice are given the same inoculum and are indicated with a different color and ID label.

Parameter calibration

The parameter estimate of the scaling factor placed on both knp,max and kmp,max is 0.227 (2.77 %). This means that the maximum phagocytosis rate is 1.895 CFU/mL/h for neutrophils and 1.766 CFU/mL/h for monocytes.

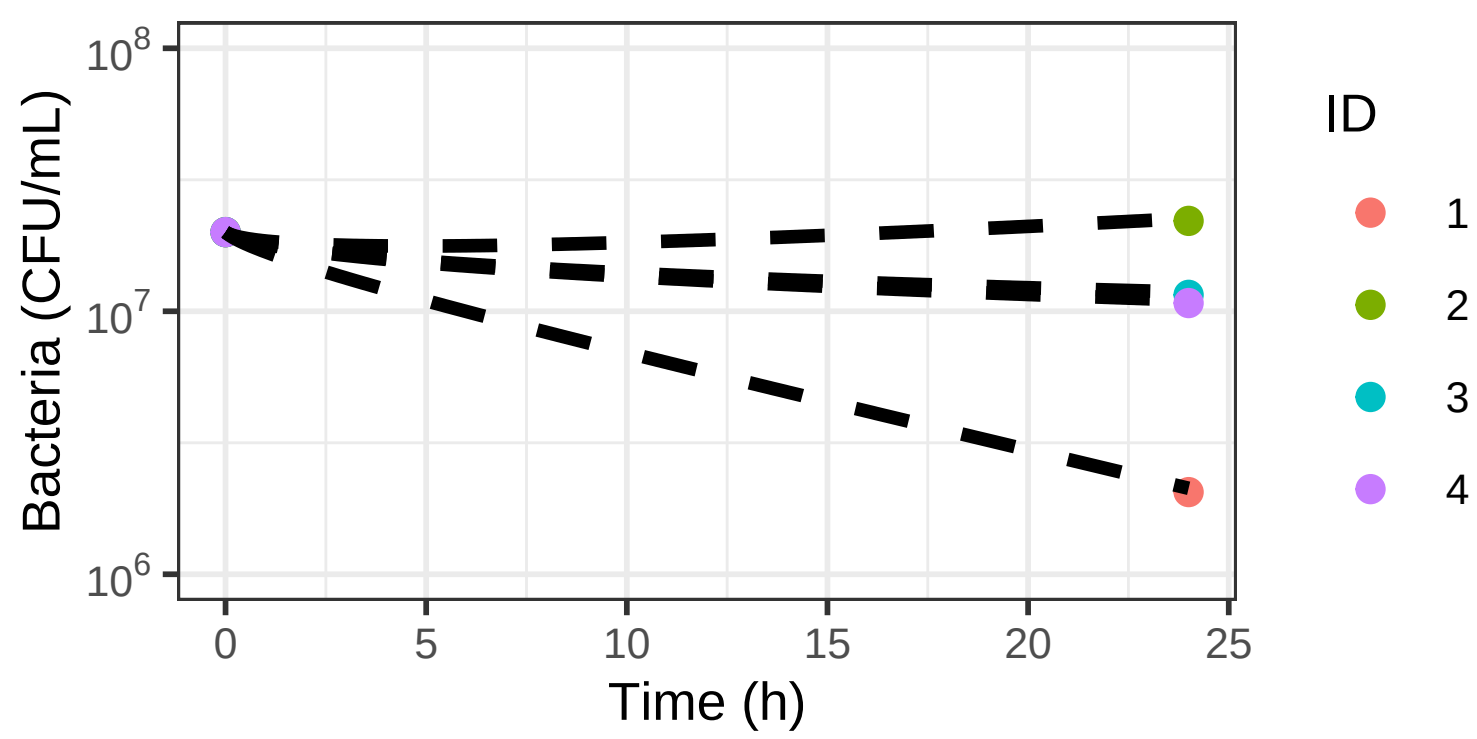

Figure 2: Visualisation of parameter fit on *in vivo* data. The four mice are given the same inoculum and are indicated with a different color and ID label. The dashed black line is the simulation of the model with the calibrated parameters.
